# Pectin-derived oligogalacturonides shape mutualistic interactions between *Bacillus* and its host plant

**DOI:** 10.1093/ismejo/wraf232

**Published:** 2025-10-18

**Authors:** Farah Boubsi, Adrien Anckaert, Anthony Argüelles-Arias, Marc Ongena

**Affiliations:** Microbial Processes and Interactions, TERRA Teaching and Research Center, University of Liège—Gembloux Agro-Bio Tech, Gembloux 5030, Belgium; Microbial Processes and Interactions, TERRA Teaching and Research Center, University of Liège—Gembloux Agro-Bio Tech, Gembloux 5030, Belgium; Microbial Processes and Interactions, TERRA Teaching and Research Center, University of Liège—Gembloux Agro-Bio Tech, Gembloux 5030, Belgium; Microbial Processes and Interactions, TERRA Teaching and Research Center, University of Liège—Gembloux Agro-Bio Tech, Gembloux 5030, Belgium

**Keywords:** *Bacillus velezensis*, plant cell wall, oligogalacturonides, pattern-triggered immunity, induced, systemic resistance, plant protection, colonization, mutualism

## Abstract

Certain beneficial bacteria of the root-associated microbiome such as *Bacillus velezensis* protect plants against diseases and are promising biocontrol agents exploited in sustainable agriculture. Unveiling the molecular dialogue governing mutualistic interactions between these beneficials and their host is essential to better understand their ecological behavior and to optimize their use as bioprotectants. However, the chemical diversity and functionality of mediators involved in this interkingdom crosstalk remain largely unexplored. In this study, we uncover a strategy by which *B. velezensis* exploits the root cell wall polymer pectin to prime its host for enhanced resistance against phytopathogens and to ensure a safe environment enabling its efficient root establishment. Thanks to the activity of its two conserved pectinolytic enzymes, the bacterium generates a specific pattern of short oligogalacturonides that act as efficient triggers of plant systemic defense against leaf pathogens. Moreover, these oligomers induce only weak immune responses in root cells and dampen local defense reaction in response to the perception of the bacterium itself. Our data emphasize the key role of short oligogalacturonides as mediators in the intricate interplay between plants and their bacterial associates, providing new insights into the mechanisms that enable beneficial bacteria to coexist with their host plant.

## Graphical Abstract

**Graphical Abstract:**
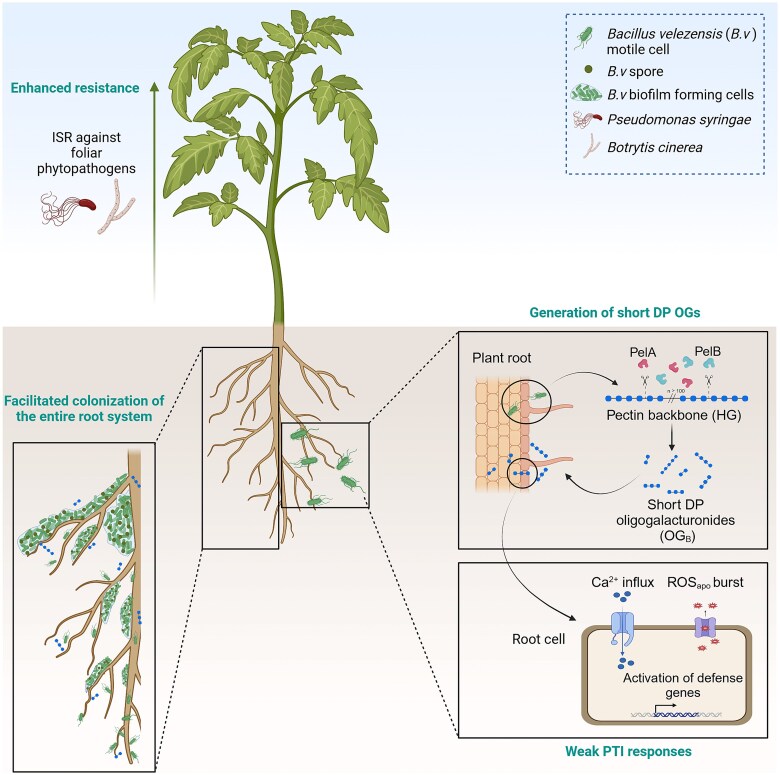

## Introduction

In metazoans, microbial symbionts are essential to ensure a wide range of physiological functions such as digestion, development of immune system, or protection against pathogens that are integral to the host's ability to thrive in diverse environments [1]. Similarly, plants establish mutualistic relationships with microbes to enhance their fitness. Root-associated microbiome comprises beneficial microbes, including symbiotic rhizobia and mycorrhizal fungi enhancing nutrient acquisition, as well as commensals and mutualists that contribute to host defense against pathogens although not forming symbioses *sensu stricto* [2, 3]. Among plant mutualists, members of the *Bacillus subtilis* complex have gained substantial attention especially for their plant protective effects [4, 5].

Within this complex, the species *Bacillus velezensis* (*B.v*) constitutes one of the most promising biocontrol agent having shown favorable impacts on plant health both *in vitro* and in field trials [6–9]. The efficacy of this species as a biocontrol agent is mainly due to its ability to produce a wide spectrum of bioactive secondary metabolites (BSMs) with a high diversity of chemical structures and functions associated. BSMs contribute to disease suppression through distinct mechanisms such as direct antagonism of microbial pathogens, competition for space and nutrients, and elicitation of induced systemic resistance (ISR) in the host plant [5]. ISR is a preconditioning of the plant defenses by its exposure to a stimulus, leading to an enhanced and/or accelerated activation of defense responses in distal tissues in response to future attacks of microbial pathogens, nematodes, or insects [10]. Among the BSMs synthesized by *B.v*, cyclic lipopeptides including surfactin, have emerged as effective ISR inducers across diverse pathosystems [11–13].

Distinguishing self from non-self is a key factor enabling plants to initiate defense mechanisms and deal with microbial invasions. The first line of plant innate immunity called pattern-triggered immunity (PTI), is activated following the perception of specific molecular patterns by cell surface pattern recognition receptors (PRRs). Upon binding with their ligands, PRRs form complexes with their co-receptor, which triggers the phosphorylation of downstream substrates that initiates a signaling cascade involving oxidative burst, calcium influx, Mitogen-activated protein kinase cascade, and hormone signaling activation [14]. These patterns such as flagellin, peptidoglycan, or chitin can be derived from microbes (i.e. Microbial-associated molecular patterns, MAMPs) and are not exclusive to pathogens [15]. They can also be displayed by beneficial microorganisms, which could hinder their ability to attach to the root surface and impair their fitness. Therefore, the successful colonization of host plants by non-symbiotic beneficial rhizobacteria relies on the acquisition of specific traits associated with the root-associated lifestyle and the establishment of a chemical dialogue with the host plant. This dialogue involves the ability of the bacterium to modulate and counteract host immune responses through spatial evasion; higher tolerance to plant toxic compounds; MAMPs divergence, degradation, sequestration, or modification [16, 17]. Despite the extensive research on the immune evasion strategies employed by pathogens, the mechanisms underlying the modulation of immune responses by commensals and mutualists still warrant further investigation [17].

In addition to the recognition of MAMPs, plants can also respond to Damage-associated molecular patterns (DAMPs) such as oligogalacturonides (OGs), released from the plant itself upon direct wounding or pathogen infection [18]. OGs of high degree of polymerization (DP), containing 10 to 15 galacturonic acid residues (OG_DP10-15_), are among the most extensively studied DAMPs capable to trigger plant immunity [19–23]. These oligomers are released from the partial degradation of pectic homogalacturonan (HG), a primary component of the plant cell wall and middle lamella, through the activity of microbial pectin/pectate lyases and polygalacturonases [24]. These pectinolytic enzymes, commonly produced by necrotrophic pathogens, are pivotal to release nutrients from the cell wall and thereby to facilitate the infection process [25, 26].

The interaction between beneficial microorganisms, such as *B.v*, and their host plant is characterized by a complex chemical cross-talk that involves the exchange of diffusible signaling molecules comprising amino acids, organic acids, sugars, and phytohormones [27, 28]. Whereas many of these compounds have been identified in plant root exudates, much remains to be discovered about the nature and diversity of the molecules that shape and influence this interaction. In this context, the perception of plant cell wall polymers play a pivotal role that can impact the fitness of beneficial microorganisms in the rhizosphere [29–32]. We recently showed that *B.v* modulates key developmental traits in response to HG. Specifically, the bacterium stimulates the synthesis of surfactin, increases biofilm formation, and accelerates its sporulation dynamics [32]. Moreover, this HG-mediated increase in *B.v* fitness was facilitated by two HG-degrading enzymes, namely PelA and PelB, constituting its very limited arsenal of pectin-degrading enzymes [32]. However, the role of the degradation products of these enzymes in the molecular dialogue occurring between the beneficial bacteria and the host plant, as well as their bioactivity in relation to plant immunity still remain unexplored.

In the present study, we investigated the outcomes of the PelA/B-mediated interaction between *B.v* and its host plant, focusing on the role of OGs generated by its two pectinolytic enzymes in this interkingdom crosstalk. Inspired by the immunogenic activity of OGs released by pathogens [19, 33], we wanted to study the possible role of OGs released by the beneficial rhizobacterium in plant immune activation. Our data show that these specific compounds play a dual role as they stimulate systemic resistance to pathogens but also attenuate local immune responses at the root level, contributing to the establishment of a mutualistic relationship between *B.v* and its host.

## Material and methods

### Plant material and growth conditions

Seeds of *Solanum lycopersicum* var. Moneymaker (Sluis Garden, Holland) and *Arabidopsis thaliana* ecotype Columbia (Col-0) were surface sterilized with 75% ethanol for 2 min, immersed in 20% bleach (NaOCl, 12%) under shaking for 10 min (tomato) or 6 min (*Arabidopsis*), and rinsed three times with sterile water. Seeds were then pregerminated and grown for 1 or 2 weeks in square Petri dishes containing half-strength Murashige and Skoog medium including vitamins (MS_1/2_, Duchefa Biochemie, The Netherlands) and supplemented with 1% (w/v) sucrose and 14 g/l agar. For all experiments, tomato and *Arabidopsis* plants were grown at 22°C under a daily photoperiod of 16 h and 12 h (100 *μ*mol s^−1^ m^−2^), respectively.

### Bacterial growth conditions

Cultures of *B. velezensis* GA1 (*B.v*) and its mutant *ΔpelAΔpelB* were initiated from overnight precultures realized in half-strength root exudate-mimicking medium (RE_1/2_) [34] pH 7. Cells were centrifuged (6000 rpm, 10 min), washed twice with sterile phosphate-buffered saline (PBS), and inoculated in RE_1/2_ at OD_600_ of 0.02. Depending on the experiment, low methylesterified galacturonate polysaccharide (Elicityl, France), referred to as HG was added at 0.1% (w/v) to RE_1/2_. Cultures were incubated at 26°C with shaking (160 rpm) for 24 h.

### Generation of oligogalacturonides produced by *Bacillus velezensis*

OG produced by *B.v* (OG_B_) were generated from the reaction of a PelA/B enriched cell-free culture supernatant (CFCS) of *B.v* Δ*sfp* mutant unable to synthesize lipopeptides and polyketides, on HG as substrate as previously described [32]. The resulting degradation products were analyzed by UPLC-qTOF MS and lyophilized as described in the same study.

OGs resulting of the activity of the Δ*sfp* CFCS enriched in PelA/B on tomato and *Arabidopsis* root tissues were identified as follows. Two-week-old tomato or *Arabidopsis* seedlings were grouped and their roots (around 80 mg fresh weight) were immersed in a 6-wells microplate with 6 ml of 50 mM Tris–HCl buffer (pH 8) and 25% (v/v) filter-sterilized Δ*sfp* CFCS in each well. The plate was incubated at 30°C under shaking (100 rpm) for 24 h. Samples of 100 *μ*l were taken at different time points and OGs profile over time was analyzed by UPLC-qTOF MS as described above.

### Systemic immunity activation assay

For *Arabidopsis*, one-week-old seedlings were transferred to seed holders of Araponics systems (Araponics, Belgium) filled with 0.7% (w/v) agar and were grown hydroponically for 3 weeks in nutrient solution (0.25% (v/v) FLORAMICRO, 0.25% (v/v) FLORABLOOM, 0.25% (v/v) FLORAGRO; General Hydroponics). Plants were then placed individually for one additional week into 50 ml falcons covered with aluminum foil containing the same solution. For tomato plants, 2-week-old seedlings were directly transferred into the 50 ml falcons and were grown for 3 weeks. Plant roots were then treated for 5 days with *B.v* or Δ*pelA*Δ*pelB* cells, or overnight with OG_B_ (50 *μ*g/ml) or CFCS from either strain in nutrient solution. *Bacillus* cultures were grown for 24 h as described in “Bacterial growth conditions”. Final OD_600_ were adjusted to 1, cultures were centrifuged (6000 rpm, 10 min), and either filtered under 0.22 *μ*m PTFE filter (for CFCS treatment) or cells were washed twice in PBS and resuspended to OD_600_ of 1 in nutrient solution (for treatment with cells). After treatment, leaf disks (5 mm Ø) were collected from the fourth or sixth leaf (*Arabidopsis*) or from the third or fourth leaf (tomato) of each plant. Apoplastic reactive oxygen species (ROS_apo_) production was then quantified upon elicitation with chitin, Flg22, or water as control as detailed below.

### Measurement of apoplastic reactive oxygen species production

ROS_apo_ burst was measured *via* a luminol-based chemiluminescence assay as previously described [35]. For *Arabidopsis*, roots from 2-week-old seedlings were grouped in sets of 10, cut into small pieces, and each set was transferred into an individual well of a white 96-well microplate (MicroFluor, Thermo Fisher Scientific, United States) with 150 *μ*l sterile water. For ROS_apo_ assay on leaves, 1 disk per well was placed in 150 *μ*l sterile water. The plate was incubated overnight in the dark at room temperature and measurement of ROS_apo_ production was performed using a Spark Tecan multiplate reader (Tecan, Switzerland). Background luminescence was recorded every minute for 15 min before adding 1 *μ*l of 5 mg/ml OG_DP10-15_ (Elicityl, France), 5 mg/ml OG_B,_ 100 *μ*m Flg22 (Eurogentec, Belgium), 10 mg/ml chitin (Sigma, United States), or water as control. Luminescence was then measured every minute for 60 min and results were expressed as luminescence or luminescence fold increase calculated as follows:


$$\text{Luminescence} \ (RLU)=L-{L}_0 \ \text{Luminescence} \ fold\ increase=\left(\frac{L-{L}_0}{L_0}\right)$$

Where L represents the luminescence measured at each time point and L_0_ represents the baseline luminescence, calculated as the average background luminescence before addition of the elicitor.

ROS_apo_ was also quantified in *Arabidopsis* roots pretreated with OG_B_ before flagellin elicitation. Two-week-old seedlings were grouped by 10 in a 12-well microplate filled with liquid MS_1/2_ for 24 h. Seedling roots were then treated overnight with MS_1/2_ supplemented or not with 50 *μ*g/ml OG_B_. Roots were subsequently washed with MS_1/2_, cut into small pieces, and incubated into a white 96-well microplate as describe above before measurement of ROS_apo_ production upon elicitation with 1 *μ*l of 100 *μ*m ultrapure *B. subtilis* flagellin (InvivoGen, United States).

### Induced systemic resistance against *Botrytis cinerea*

Tomato plants were grown and their roots were pretreated with OG_B_ (50 *μ*g/ml) or nutrient solution as control as described in “systemic immunity activation (SIA) assay”. *Botrytis cinerea* strain MUCL 43839 was cultured on solid PDA for 15 days in the dark at room temperature. Spores were then collected in a germination solution (1.75 g/l KH_2_PO_4_, 0.74 g/l MgSO_4_, 4 g/l glucose, 0.02% (w/v) Tween20), filtered through a sterile gauze, quantified, and adjusted to a concentration of 5 × 10^5^ spores/ml. The spore suspension was then incubated overnight under shaking at 26°C to allow the pregermination of the spores. Infection was performed by applying a 5 *μ*l drop of *B. cinerea* spore suspension onto three leaflets of the third and fourth leaves. Infected plants were incubated at 19°C with a HR of 65% under a 16 h/8 h day/night cycle (100 *μ*mol s^−1^ m^−2^). The number of spreading lesions and their area were measured by color thresholding using ImageJ Fiji software [36] 5 days post-infection.

### Induced systemic resistance against *Pseudomonas syringae*

Tomato and *Arabidopsis* plants were grown and their roots were pretreated as described above. *Pseudomonas syringae* DC3000 was cultured in liquid King’s B (KB) medium at 28°C for 24 h with shaking (160 rpm) as previously described [37]. The culture was then centrifuged (6000 rpm, 10 min), cells were washed twice in 10 mM MgSO_4_, and OD_600_ was adjusted to 0.2 in 10 mM MgSO_4_ supplemented with 0.02% (w/v) Tween20. Infection was performed as previously described [38] by pressure infiltration of 1 ml of the bacterial suspension onto the abaxial surface of three leaflets from the third and fourth leaves (tomato) or three leaves (fourth to sixth) for *Arabidopsis*. Infected plants were incubated at 22°C in transparent Sun Bags (Sigma, United States) under a 16 h/8 h day/night cycle (100 *μ*mol s^−1^ m^−2^) for 4 days (tomato) or 3 days (*Arabidopsis*). Then, one leaf disk (5 mm Ø) was cut from each infected leaflet or leaf. All disks from a single leaf (tomato) or from an individual plant (*Arabidopsis*) were pooled together and transferred into 1 ml of 10 mM MgSO₄ solution [38]. Tissues were ground with an Eppendorf pestle, vortexed, and diluted in 1:10 series. Dilutions were plated on solid KB supplemented with 50 *μ*g/ml rifampicin and incubated at 28°C for 48 h. Total CFUs were counted and results were expressed as CFU/cm^2^ of leaf disk.

### Calcium influx measurement

Calcium influx measurement was assessed through fluorescence using the *A. thaliana* UBQ10::GCaMP3 reporter line [39]. Five-day-old seedlings were grown in round Petri dishes (94 mm Ø) containing 10 ml sterile MS_1/2_ supplemented with 1% (w/v) sucrose and 1% (w/v) agarose. Fluorescence signals (470 nm_Ex_/535 nm_Em_) were monitored as previously described [39], using a Nikon SMZ1270 stereomicroscope (Nikon, Japan). To establish the baseline fluorescence, a 2 min video (0.5 fps) was recorded before elicitation for each root. Then, 10 *μ*l of OG_DP10-15_ (50 *μ*g/ml), OG_B_ (50 *μ*g/ml), or water were applied on the root tip of the seedlings. Calcium influx dynamics were recorded for 6 min (0.5 fps) and fluorescence signals were analyzed in a region of interest (ROI) located at 0.26 cm from the root tip, then normalized using the equation:


$$ \frac{\Delta F}{F_0}=\frac{\left(F-{F}_0\right)}{F_0} $$

where F represents the fluorescence measured at each time point and F_0_ represents the baseline fluorescence calculated as the average of F over the first 2 min prior to treatments.

Images at 0, 60, and 120 s were extracted and pseudo-colored with fixed LUTs. Data and image processing were performed using NIS-Elements imaging software (Nikon, Japan).

### Seedling growth inhibition

Five-day-old *Arabidopsis* seedlings were transferred individually in a 48-well microplate with 500 *μ*l MS_1/2_ supplemented or not with 200 *μ*g/ml OG_DP10-15_ or 200 *μ*g/ml OG_B_ and were allowed to grow for 10 days. Each seedling was then gently blotted on absorbent paper before being weighed on an analytical scale.

### RNA extraction

Twelve-day-old *Arabidopsis* seedlings were transferred individually in a 12-well microplate filled with MS_1/2_. After 10 days, fresh medium was added to the wells. After 24 h, seedlings were grouped by six and their roots were treated in MS_1/2_ supplemented with 50 *μ*g/ml OG_DP10-15_, 50 *μ*g/ml OG_B,_ or water as control for 1 h or 3 h. Roots were then flash-frozen in liquid nitrogen and ground with an Eppendorf pestle. RNA was extracted using the Plant RNeasy Plant Mini Kit (Qiagen, United States) following the manufacturer’s protocol with an additional step of treatment with RNAse-Free DNase Set (Qiagen, United States). For bacterial samples, RNA extractions from *B.v* cells were performed using the NucleoSpin RNA Kit (Macherey Nagel, Germany), following the manufacturer’s protocol for Gram-positive bacteria. RNA concentration and purity were assessed for both plant and bacterial samples with the UV–vis spectrophotometer NanoDrop 2000 (Thermo Fisher Scientific, United States).

### RT-qPCR analysis

RT-qPCR reactions were performed as previously described [32] using the Luna Universal One-step RT-qPCR Kit (New England Biolabs, United States) in a volume of 20 *μ*l. The transcription level of genes of interest was analyzed using the mathematical model proposed by Pfaffl [40]. For *B.v* samples, *gyrA* was used as a housekeeping gene. For *Arabidopsis* samples, the expression stability of six housekeeping genes (*UBQ10, ACT7, TUB6, EF1Α, EIF4A,* and *UBQ5*) was assessed under our experimental conditions using the web-based tool RefFinder [41]. Based on comprehensive ranking, *EIF4A* was identified as the most suitable reference gene for normalization. All the RT-qPCR primers used in this study are listed in supplementary Table S1.

### Microscopy for root colonization

Four-week-old *Arabidopsis* seedlings grown in round Petri dishes (94 mm Ø) filled with 10 ml sterile MS_1/2_ supplemented with 1% (w/v) agarose were elicited with 5 *μ*l OG_DP10-15_ (50 *μ*g/ml), OG_B_ (50 *μ*g/ml), or water at the tip of the primary root. To assess the preferential colonization of primary versus lateral roots, *Arabidopsis* seedlings were grown for 2 weeks on sterile MS_1/2_ supplemented with 1% (w/v) Bacto agar (Sigma, United States) and 0.01% (w/v) MES (Sigma, United States) before being treated as described. Seedlings were then inoculated 0.5 cm below the start of the hypocotyl with 2 *μ*l of a cell suspension of mCherry-tagged *B.v* at OD_600_ 0.1 prepared as described above. Petri dishes were sealed with parafilm and incubated for 4 days at 22°C under a 12 h photoperiod (100 *μ*mol s^−1^ m^−2^). Composite images of bacterial colonization were acquired by epifluorescence microscopy (560 nm_Ex_/630 nm_Em_) using a Nikon Ti2-E inverted microscope (Nikon, Japan) equipped with a × 20/0.45 NA S Plan Fluor objective lens (Nikon, Switzerland) and a Nikon DS-Qi2 monochrome microscope camera. A lumencor sola illuminator (Lumencor, USA) was used as source of excitation (500 ms exposure time). Acquisitions were processed with the NIS-Element AR software (Nikon, Japan) to measure the area colonized by the bacteria and the sum of fluorescence intensity in these areas *via* color thresholding.

To measure the area colonized specifically on primary root versus lateral roots, composite images were captured by epifluorescence stereomicroscopy using a Nikon SMZ1270 stereomicroscope (Nikon, Japan) equipped with a DS-F 2.5 F-mount adapter and an ED Plan Apo 1×/WF objective (Nikon, Switzerland). The epifluorescence images were captured using a C-FLL-C mCherry filter cube (570 nm_Ex_/645.5 nm_Em_) (Nikon, Japan) and an OCC illuminator (500 ms exposure time). Primary and lateral root systems were then delimited within specific ROIs using the NIS-Element AR software (Nikon, Japan), in which the area colonized was quantified by color thresholding.

Composite images of the entire root system of each seedling were captured by bright-field stereomicroscopy. The stereomicroscope was equipped with a Nikon DS-Qi2 monochrome microscope camera and a DS-F 1× F-mount adapter and used with an ED Plan Apo 1×/WF objective (Nikon, Switzerland) and an OCC illuminator (2 ms exposure time). Total root area was measured *via* color thresholding using ImageJ Fiji software. Root colonization, colonization efficiency, and relative colonization index were then calculated for each seedling as follows:


\begin{align*} &Root\ colonization\ \left( fluorescent\ pixel/c{m}^2\right)\\&\quad=\frac{\sum \left( sum\ fluorescence\ in tensities\ in\ each\ region\ colonized\right)}{Total\ root\ area} \end{align*}

$$ Colonization\ efficiency\ \left(\%\right)=\left(\frac{Root\ area\ colonized}{Total\ root\ area}\right)\times 100 $$

$$ Relative\ colonization\ index=\frac{\left(\frac{Area\ colonized\ on\ the\ primary\ root}{Total\ area\ of\ the\ primary\ root}\right)}{\left(\frac{Area\ colonized\ on\ lateral\ roots}{Total\ area\ of\ lateral\ roots}\right)} $$

### Quantification of spore population

To quantify spore population of mCherry-tagged *B.v* on roots of *Arabidopsis* seedlings, roots were separated from the aerial part and placed in Eppendorf tubes with glass beads and 1 ml PBS supplemented with 0.1% (w/v) Tween20. Tubes were vortexed for 5 min and bacterial suspensions were diluted in 1:10 series. Dilutions were then heated (85°C, 10 min), plated on solid LB supplemented with 5 *μ*g/ml chloramphenicol, and incubated at 30°C overnight for colony counting. Spore population was normalized against the corresponding seedling total root area and results were expressed as CFU/cm^2^ of root.

### Statistical analyses

Statistical analyses were performed on GraphPad Prism 9. Analysis of variance was executed on each data set and statistical differences between means were assessed either through two-tailed unpaired Student’s t-test or Dunnett’s multiple comparisons (α = .05). The number of biological replicates used for each experiment and *P* values are indicated in the legends of the corresponding figures.

## Results

### Oligogalacturonides generated by Pel enzymes of *Bacillus velezensis* prime plant systemic resistance

**Figure 1:**
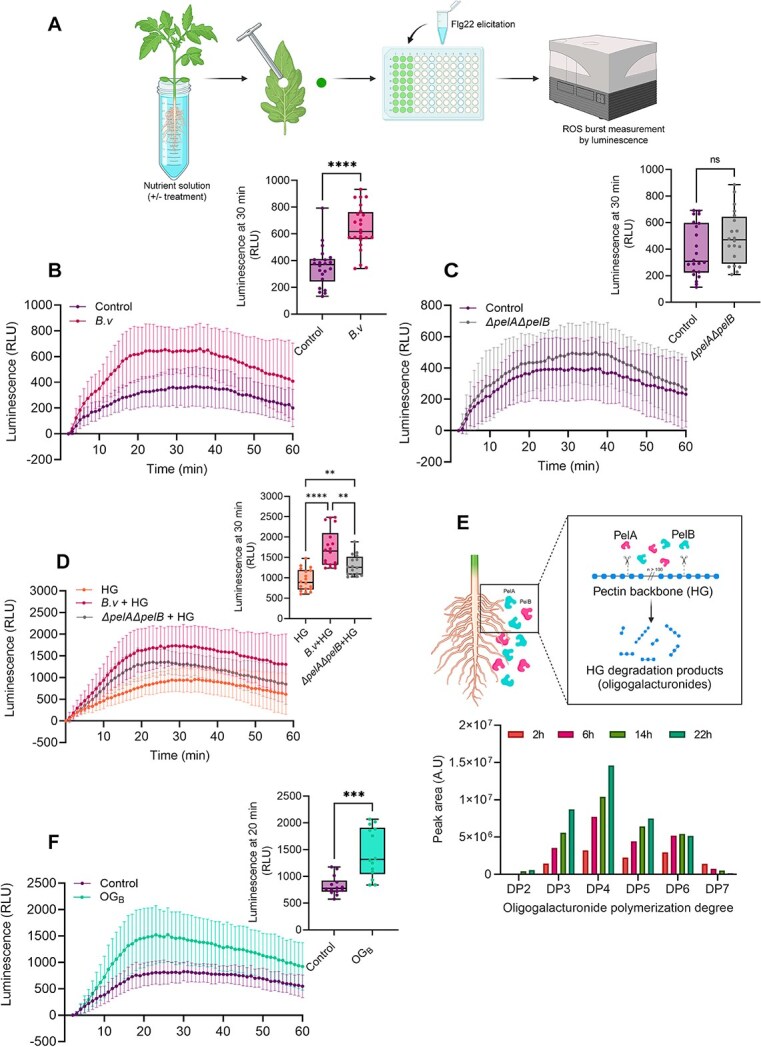
Systemic immunity is activated by pectin degradation products generated by *B.v.* (A) Schematic representation of the SIA assay (created with biorender.com). (B and C) Time-course measurement of ROS_apo_ burst in leaves of tomato plants whose roots were pretreated or not with *B.v* cells (B) or with *B.v ΔpelAΔpelB* mutant cells (C) (mean ± SD, *n* = 22–24 biological replicates with one disk per individual plant (B) or *n* = 20–22 biological replicates with one disk per individual plant (C), three independent experiments). Boxplots show the quantification of the maximal ROS_apo_ production measured in each condition (t-test; ns, non-significant; ^****^, *P* < .0001). (D) Time-course measurement of ROS_apo_ burst in leaves of tomato plants whose roots were pretreated or not with 24 h CFCS of *B.v* or *ΔpelAΔpelB* mutant strain in presence of 0.1% HG (mean ± SD; *n* = 16–18 biological replicates with one disk per individual plant, two independent experiments). Boxplot shows the quantification of the maximal ROS_apo_ production measured in each condition (Dunnett’s multiple comparisons test; α = .05; ^**^, *P* < .01; ^****^, *P* < .0001). (E) Kinetics and UPLC-qTOF-MS characterization of OGs produced over time from tomato roots by the activity of cell free PelA-PelB enriched extract from a culture supernatant of *B.v*. (F) Time-course measurement of ROS_apo_ burst in leaves of tomato plants whose roots were pretreated or not with 50 *μ*g/ml OGs produced by *B.v* (OG_B_) (mean ± SD; *n* = 14 biological replicates with one disk per individual plant, two independent experiments). Boxplot shows the quantification of the maximal ROS_apo_ production measured in each condition (t-test;^***^, *P* < .001). All boxplots of the panel encompass the first and third quartiles, the whiskers extend to the minimum and maximum points, and the midline indicates the median.

We evaluated the potential of *B.v* inoculated on roots of 4-week-old tomato plants to prime immune responses in leaves upon perception of the well-described MAMP flagellin [15] in a process referred to as “Systemic Immune Activation” (SIA). More specifically, we monitored the burst of ROS_apo_ in leaf tissues in response to treatment with the Flg22 epitope from pathogenic *Pseudomonas* (Fig. 1A). Results showed a significantly higher SIA response in leaves from plants whose roots were colonized by *B.v* compared to non-inoculated control plants (Fig. 1B). Conversely, such enhanced ROS_apo_ response was not observed in leaves from plants inoculated with the *ΔPelAΔpelB* mutant strain of *B.v* unable to degrade pectin (Fig. 1C). This indicates that SIA might be mediated by OGs released from pectin of the plant cell wall by *B.v* through the activity of its two pectate lyases, PelA and PelB, during the process of root colonization. However, we also observed that the *ΔPelAΔpelB* mutant exhibits a reduced colonization ability compared to the wild-type strain (Supplementary Fig. S1). Because lower bacterial population on the plant roots could directly affect the amounts of other immunogenic compounds produced by *B.v*, such as lipopeptides, it was necessary to decouple the SIA effect from the colonization ability of the two strains. Therefore, we next performed SIA assays on tomato plants of the same developmental stage whose roots were pretreated with CFCSs of either *B.v* or *ΔpelAΔpelB* mutant, both grown in root exudates mimicking medium supplemented with HG and adjusted to the same final OD_600_. Data showed a reduced ROS_apo_ burst in leaves from plants pretreated with *ΔpelAΔpelB* cell-free supernatant in which OGs are not generated compared to those pretreated with the wild-type strain cell-free supernatant (Fig. 1D). This further supports the substantial contribution of HG degradation products in the global potential of the strain to stimulate systemic defense response.

To characterize these OGs, we analyzed their DP profile when generated by *B.v.* from native pectin found in root tissues. To that end, tomato roots were incubated with a PelA/B-enriched extract obtained through ultrafiltration of cell-free *B.v* culture supernatant and OGs profiling over time was performed by UPLC qTOF-MS. The degradation products detected were predominantly short (DP 2-7), with an average DP of 4 from the onset of the reaction (Fig. 1E). A similar OGs profile was also observed when using commercial HG as substrate (Supplementary Fig. S2), and this extract was considered representative of pectin degradation products generated by *B.v*, hereafter referred to as OG_B_. These OG_B_ were subsequently tested for SIA upon root treatment at 50 *μ*g/ml, which is the minimal active working concentration of OGs commonly reported [22, 42–44]. Plants pretreated with OG_B_ displayed a significantly higher ROS_apo_ response to Flg22 in leaves (Fig. 1F), confirming their potential for priming the plant immune system.

**Figure 2:**
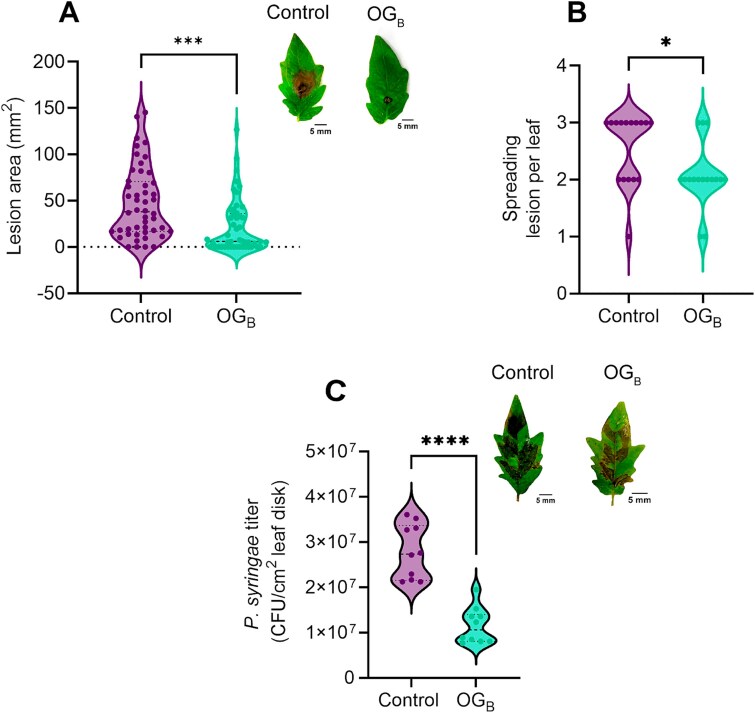
OG_B_ enhance plant systemic resistance against phytopathogens. (A and B) Quantification of the lesion area (A) and number of spreading lesions (B) observed on leaves from tomato plants whose roots were pretreated or not with 50 *μ*g/ml OG_B_ 5 days post-infection with a spore suspension of *B. cinerea* (5x10^5^ spores/ml) (*n* = 48, six leaflets infected per plant from eight individual plants (A) or *n* = 16, three leaflets infected from two leaves per plant from eight individual plants (B); t-test; ^*^, *P* < .05; ^***^, *P* < .001). Images are representative pictures of spreading lesions observed on tomato leaflets 5 days post-infection with *B. cinerea*. (C) Bacterial titer of *P. syringae* pv. tomato DC3000 on leaves form tomato plants whose roots were pretreated or not with 50 *μ*g/ml OG_B_ 4 days post-infiltration (*n* = 10, two leaves infected per plant from five individual plants; t-test; ^****^, *P* < .0001). Images are representative pictures of OG_B_-induced resistance of tomato plants against *P. syringae* pv. tomato DC3000 4 days post-infiltration. All violin plots of the panel show the distribution and density of the data. The width of the violin indicates the density at different values, with thicker sections representing higher data density. The central bold dashed line represents the median and the thin dashed lines indicate the interquartile range.

Our results prompted us to evaluate whether OG_B_ also retain the potential to protect plants *via* ISR when perceived at the root level. Pretreatment of tomato plant roots with OG_B_ led to a reduction in the size and number of lesions measured on the leaves drop-inoculated with spores of the necrotrophic fungus *Botrytis cinerea* compared with the untreated plants, resulting to a disease reduction of ~72% (Fig. 2A and B). OG_B_ were also tested for triggering systemic resistance against infection caused by the hemibiotrophic bacterium *P. syringae* DC3000 inoculated *via* pressure infiltration. Results showed that the bacterial titer was significantly reduced in leaves of plants pretreated with OG_B_ compared to non-pretreated control plants (Fig. 2C).

### OG_B_ perception is associated with weak local immune responses

The contribution of *pel* enzymes to the OGs profile generated from tomato roots, as well as their role in tomato root colonization were also evident in *A. thaliana* (hereafter referred to as *Arabidopsis*) (Supplementary Figs S3 and S4), suggesting that the plant can serve as a suitable model for further investigations of immune-related processes. Moreover, similar to tomato, *Arabidopsis* plants whose roots were pretreated with 50 *μ*g/ml OG_B_ displayed enhanced SIA responses when challenged with chitin or flg22 to simulate the presence of *B. cinerea* or *P. syringae*, respectively (Supplementary Fig. S5A and B). Accordingly, OG_B_ pretreatment also conferred increased systemic resistance against both pathogens (Supplementary Fig. S5C and D). Building on these observations, we used *Arabidopsis* as a model plant to investigate typical PTI-associated immune responses triggered by OG_B_ in root tissues, using OG_DP10-15_ as a reference DAMP known for its strong PTI-inducing activity across several plant species [22, 44–47].

**Figure 3:**
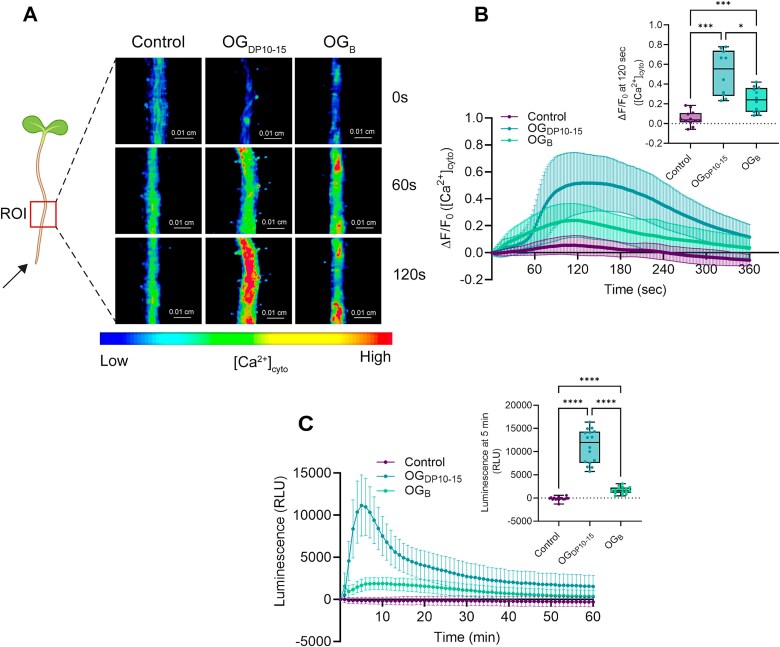
OG_B_ trigger weak PTI responses. (A) Representative pseudo-colored images of time-course changes in [Ca^2+^]_cyt_ in the roots of *Arabidopsis* UBQ10::GCaMP3 reporter line into the ROI (red square) at a distance of 0.26 cm from the root tip after application of 50 *μ*g/ml OG_DP10-15_, 50 *μ*g/ml OG_B_, or water as control at the root tip (black arrow). (B) Quantification of the [Ca^2+^]_cyt_ signature in roots of *Arabidopsis* UBQ10::GCaMP3 reporter line in the ROI (mean ± SD; *n* = 10–12 biological replicates, two independent experiments). Boxplot shows the quantification of the maximal [Ca^2+^]_cyt_ measured in the ROI in the different conditions (Dunnett’s multiple comparisons test; α = .05; ^*^, *P* < .05; ^***^, *P* < .001). (C) Time-course measurement of ROS_apo_ production by chemiluminescence in *Arabidopsis* roots elicited with 50 *μ*g/ml OG_DP10-15_, 50 *μ*g/ml OG_B_, or water as control (mean ± SD; *n* = 16 biological replicates each containing 10 roots from 10 seedlings, two independent experiments). Boxplot shows the quantification of the maximal ROS_apo_ production measured by chemiluminescence in *Arabidopsis* roots in each condition (Dunnett’s multiple comparisons test; α = .05; ^****^, *P* < .0001). All boxplots of the panel encompass the first and third quartiles, the whiskers extend to the minimum and maximum points, and the midline indicates the median.

Calcium (Ca^2+^) influx is one of the most prevalent and early signaling event essential to orchestrate plant immune responses [48]. Dynamic changes in Ca^2+^ balance in *Arabidopsis* roots were monitored by fluorescence microscopy, using the transgenic *Arabidopsis* reporter line UBQ10::GCaMP3 [39]. Transgenic seedlings were locally elicited at the root tip either with 50 *μ*g/ml OG_DP10-15_, OG_B_, or water as control and calcium signal transmission along the roots was observed over time (Fig. 3A and Supplementary Videos S1, S2, S3). Within seconds following application, OG_DP10-15_ triggered a strong calcium response in *Arabidopsis* seedlings, which was visualized by an intense [Ca^2+^]_cyt_ increase along the root (Fig. 3B). However, the calcium signature in response to OG_B_ differed markedly from that induced by OG_DP10-15,_ with a significantly lower amplitude of [Ca^2+^]_cyt_ increase (Fig. 3B). We then tested the potential of OG_B_ to induce the production of ROS_apo_ in roots of *Arabidopsis* seedlings. Root tissues were elicited with 50 *μ*g/ml OG_DP10-15_, OG_B_, or water as control and the production of ROS_apo_ over time was monitored by luminescence using the L012-peroxidase system. In line with calcium influx measurements, OG_B_ induced only a slow and weak ROS_apo_ response, whereas OG_DP10-15_ triggered a rapid and intense burst within the first few minutes of the reaction (Fig. 3C).

**Figure 4:**
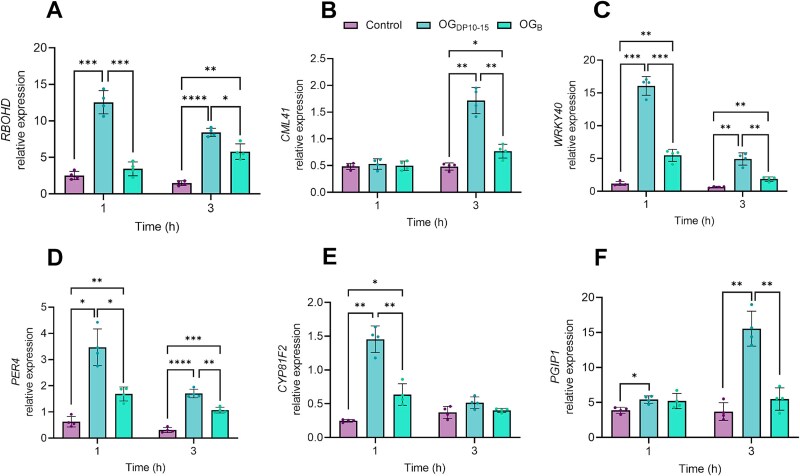
OGs-related immunity marker genes are weakly activated by OG_B_ (A–F) expression pattern of *RBOHD* (A), *CML41* (B), *WRKY40* (C), *PER4* (D), *CYP81F2* (E), and *PGIP1* (F) genes in *Arabidopsis* roots treated with 50 *μ*g/ml OG_B_, 50 *μ*g/ml OG_DP10-15_, or water as control. Expression level of each gene was normalized against the expression level of the housekeeping gene *EIF4A*. Only significant differences are displayed in the graphs (mean ± SD; *n* = 4 biological replicates each containing six roots from six seedlings; Dunnett’s multiple comparisons test; α = .05; ^*^, *P* < .05; ^**^, *P* < .01; ^***^, *P* < .001; ^****^, *P* < .0001).

To further compare the effect of OG_B_ and OG_DP10-15_ at the transcriptomic level, we measured the expression levels of a subset of immune-related genes in *Arabidopsis* roots *via* RT-qPCR 1 h and 3 h post-treatment. We selected genes that were previously identified as specifically modulated in response to OG_DP10-15_ in whole *Arabidopsis* seedlings [22, 46]. *RBOHD* gene encoding the plasma membrane NADPH oxidase RBOHD, which is predominantly responsible for ROS production [49], was significantly up-regulated by both OG_DP10-15_ and OG_B_, with an earlier and stronger induction upon OG_DP10-15_ treatment (Fig. 4A). These results are in accordance with the difference in ROS_apo_ burst amplitude observed upon treatment with both elicitors (Fig. 3C). Similarly, *CML41* encoding a calcium-binding calmodulin-like protein involved in dampening plant immune responses [22] was strongly expressed in roots treated with OG_DP10-15_ compared to those treated with OG_B_ after 3 h (Fig. 4B). The expression of *WRKY40* encoding a WRKY transcription factor responsible for the activation of jasmonic acid-dependent plant defense responses [50] as well as the anionic peroxidase encoding gene *PER4* followed the same trend at both time points (Fig. 4C and D). The expression of *CYP81F2* encoding the cytochrome P450 involved in indole glucosinolate metabolism [51] was significantly up-regulated by OG_DP10-15_ compared to OG_B_ 1 h post-treatment (Fig. 4E) whereas the expression of *PGIP1* gene coding for the polygalacturonase inhibiting protein PGIP1 was exclusively induced in *Arabidopsis* roots elicited with OG_DP10-15_ (Fig. 4F). This last result obtained in *Arabidopsis* roots contrasts with the upregulation of *PGIP1* previously reported upon treatment of whole *Arabidopsis* seedlings with OG_DP3_ [46] and may be attributed to the organ-specific regulation of gene expression [52].

**Figure 5:**
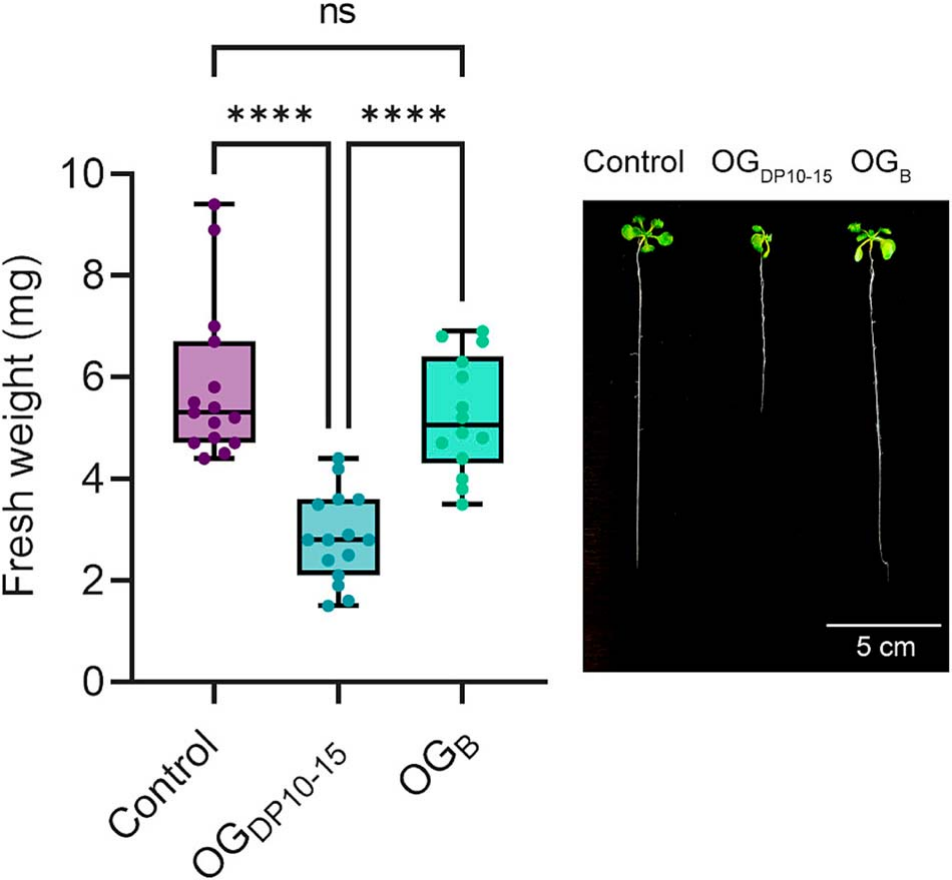
OG_B_ treatment does not impair plant growth. Fresh weight of *Arabidopsis* seedlings grown in MS_1/2_ medium as control and upon supplementation with 200 *μ*g/ml OG_DP10-15_ or 200 *μ*g/ml OG_B_ (*n* = 14–15 biological replicates, two independent experiments; Dunnett’s multiple comparisons test; α = .05; ns, non-significant; ^****^, *P* < .0001). The boxplot encompasses the first and third quartiles, the whiskers extend to the minimum and maximum points, and the midline indicates the median. The image is a representative picture of *Arabidopsis* seedlings growth inhibition in presence 200 *μ*g/ml OG_DP10-15_ or 200 *μ*g/ml OG_B_.

Plants must carefully balance their energy allocation between growth and defense mechanisms to maintain an optimal development. Strong activation of defense responses by MAMPs or DAMPs can result in adverse effects such as reduced overall growth, hypersensitivity, or cell death [53]. Considering the difference of immune response intensities between OG_B_ and OG_DP10-15_, we investigated whether OG_B_ could also have a fitness cost on the plant. Contrary to root treatment with OG_DP10-15_ that led to a significant decrease of *Arabidopsis* seedling growth, OG_B_ treatment did not exhibit such detrimental effect as shown by the similar size of seedlings compared to untreated controls (Fig. 5).

### OG_B_ dampen local immune response and facilitate root colonization by *Bacillus velezensis*

**Figure 6:**
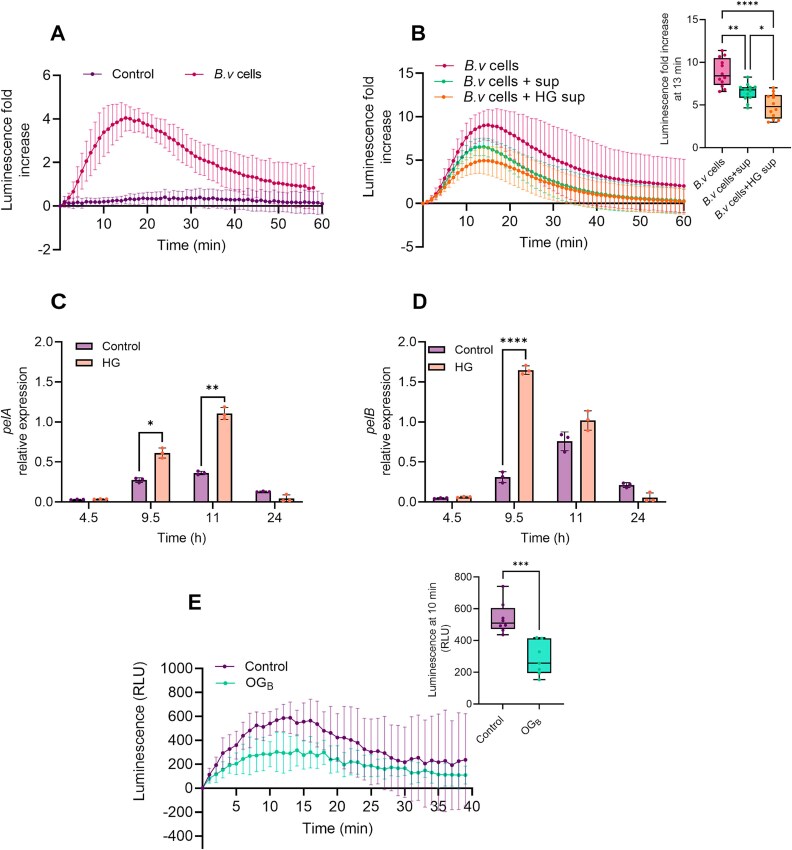
HG sensing activates *pelA* and *pelB* expression in *B.v* and HG degradation products dampen plant immune response to the bacterium. (A) Time-course measurement of ROS_apo_ burst by chemiluminescence in *Arabidopsis* roots elicited with *B.v* cells or with PBS as control (mean ± SD; *n* = 5 biological replicates, each containing 10 roots from 10 seedlings). (B) Time-course measurement of ROS_apo_ production by chemiluminescence in *Arabidopsis* roots elicited with *B.v* cells washed with PBS or with *B.v* cells resuspended in a 24 h-cell-free spent culture supernatant of *B.v* supplemented or not with 0.1% (w/v) HG (mean ± SD, *n* = 12 biological replicates, each containing 10 roots from 10 seedlings, two independent experiments). Boxplot shows the quantification of the maximal ROS_apo_ production measured in each condition (Dunnett’s multiple comparisons test; α = .05; ^*^, *P* < .05; ^**^, *P* < .01; ^****^, *P* < .0001). (C and D) Expression pattern of *pelA* (C) and *pelB* (D) genes in *B.v* cells grown in RE_1/2_ medium as control and upon supplementation with 0.1% (w/v) HG. Expression level of each gene was normalized against the expression level of the *gyrA* housekeeping gene (mean ± SD; *n* = 3 biological replicates; t-test; ^*^, *P* < .05; ^**^, *P* < .01; ^****^, *P* < .0001). (E) Time-course measurement of ROS_apo_ production by chemiluminescence in *Arabidopsis* roots pretreated or not overnight with 50 *μ*g/ml OG_B_ and subsequently elicited with 1 *μ*m pure flagellin of *B. subtilis* (mean ± SD; *n* = 7–8 biological replicates, each containing 10 roots from 10 seedlings). Boxplot shows the quantification of the maximal ROS_apo_ production measured in each condition (t-test; ^***^, *P* < .001). All boxplots of the panel encompass the first and third quartiles, the whiskers extend to the minimum and maximum points, and the midline indicates the median.

**Figure 7:**
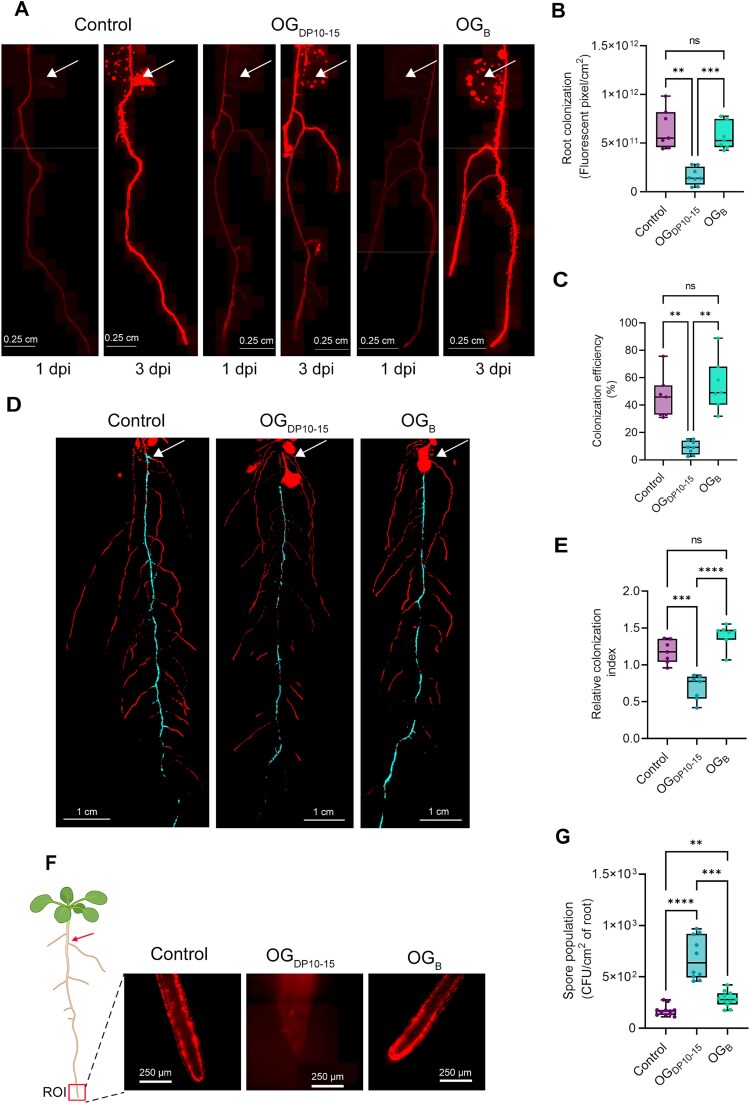
Root elicitation with OG_DP10-15_ and OG_B_ differentially shape *B.v* colonization, distribution, and spore formation. (A) Microscopic composite pictures of mCherry-tagged *B.v* colonization along roots of *Arabidopsis* seedlings elicited at the root tip with 50 *μ*g/ml OG_B_, 50 *μ*g/ml OG_DP10-15_, or water as control. Images were taken 1 and 3 dpi. White arrows indicate the inoculation drop of the bacterial suspension. (B) Quantification of mCherry-tagged *B.v* colonization along roots of *Arabidopsis* seedlings elicited at the root tip with 50 *μ*g/ml OG_B_, 50 *μ*g/ml OG_DP10-15_, or water as control 3 dpi (*n* = 7–8 biological replicates, three independent experiments; Dunnett’s multiple comparisons test; α = .05; ns, non-significant; ^**^, *P* < .01; ^***^, *P* < .001). (C) Quantification of the colonization efficiency of *Arabidopsis* seedling roots elicited at the root tip with 50 *μ*g/ml OG_B_, 50 *μ*g/ml OG_DP10-15_, or water as control by mCherry-tagged *B.v* 3 dpi. Colonization efficiency was calculated as the ratio between the root area colonized by *B.v* and the total area of the root system (*n* = 7–8 biological replicates, three independent experiments; Dunnett’s multiple comparisons test; α = .05; ns, non-significant; ^**^, *P* < .01). (D) Macroscopic composite pictures of mCherry-tagged *B.v* colonization along the primary root (cyan) and lateral roots (red) of *Arabidopsis* seedlings elicited at the root tip with 50 *μ*g/ml OG_B_, 50 *μ*g/ml OG_DP10-15_, or water as control. Images were taken 4 dpi. White arrows indicate the inoculation drop of the bacterial suspension. (E) Quantification of the relative colonization index of mCherry-tagged *B.v* on roots of *Arabidopsis* seedlings elicited at the root tip with 50 *μ*g/ml OG_B_, 50 *μ*g/ml OG_DP10-15_, or water as control 4 dpi (*n* = 7 biological replicates, two independent experiments; Dunnett’s multiple comparisons test; α = .05; ns, non-significant; ^***^, *P* < .001; ^****^, *P* < .0001). Relative colonization index was calculated for each seedling as the ratio between the area colonized by *B.v* on the primary root normalized by the total area of the primary root and the area colonized by *B.v* on lateral roots normalized by the total area of lateral roots. (F) Microscopic pictures of *Arabidopsis* root tip colonization by mCherry-tagged *B.v* 2 dpi after elicitation of the root tip with water as control, 50 *μ*g/ml OG_DP10-15_, or 50 *μ*g/ml OG_B_. Red arrow indicates the inoculation point of the bacterial suspension and the ROI delimits the zone of the root from which detailed microscopic pictures were captured. (G) Quantification of spore population of mCherry-tagged *B.v* 3 dpi by plate counting on roots of *Arabidopsis* elicited at the root tip with 50 *μ*g/ml OG_B_, 50 *μ*g/ml OG_DP10-15_, or water as control. (*n* = 10–11 biological replicates, two independent experiments; Dunnett’s multiple comparisons test; α = .05; ^**^, *P* < .01;;^***^, *P* < .001; ^****^, *P* < .0001). All boxplots of the panel encompass the first and third quartiles, the whiskers extend to the minimum and maximum points, and the midline indicates the median.

Beneficial bacilli harbor MAMPs similar to those of pathogens such as flagellin, which can also be recognized by the host plant to mount immune responses [17, 54]. Accordingly, we observed that washed cells of *B.v* trigger a substantial immune reaction in 2-week-old *Arabidopsis* roots by using ROS_apo_ burst as proxy (Fig. 6A). However, adding to this cell suspension a spent medium resulting from *B.v* cultivation in presence of HG led to a reduction of the ROS_apo_ burst compared to that induced either by cells alone or by cells in the presence of spent medium lacking HG (Fig. 6B). These results indicate that the host immune response mounted upon recognition of *B.v* cells could be attenuated by certain metabolites or bioactive compounds released in the supernatant during bacterial growth in presence of pectin. Additionally, both *pelA* and *pelB* genes are upregulated in *B.v* upon cultivation in presence of HG (Fig. 6C and D). Such modulation of pectin degrading gene expression implies that when the bacterium encounters pectin backbone during the process of root colonization, its degradation machinery is promptly activated, facilitating the release of OG_B_. On that basis, we wanted to evaluate the potential of OG_B_ to dampen the plant immune response to *Bacillus* itself. To do so, roots of *Arabidopsis* seedlings were pretreated overnight with OG_B_ before being elicited with 1 *μ*m pure flagellin of *B. subtilis*, which displays a Flg22 epitope homologous to the one of *B.v* (Supplementary Fig. S6). ROS_apo_ measurements showed that pretreated seedlings were less sensitive to flagellin of *Bacillus* compared with untreated control seedlings (Fig. 6E). In light of these results, we assume that promptly generating OG_B_ from pectin upon root contact allows *B.v* to counteract the activation of a strong immune response by the plant and can be interpreted as a strategy to dampen host immunity and promote root establishment. Therefore, we next wanted to assess whether the production of OG_B_ instead of pathogen-associated OG_DP10-15_ might confer a fitness advantage to *B.v.* To that end, root colonization of *Arabidopsis* seedlings by mCherry-tagged *B.v* was monitored by fluorescence microscopy over three days post-elicitation at the root tip either with OG_B_, OG_DP10-15_, or water as control_._ Microscope composite images of *Arabidopsis* root system showed that treatment with OG_DP10-15_ markedly inhibited root invasion by *B.v*, whereas bacterial colonization of seedlings treated with OG_B_ was robust and similar to control conditions. (Fig. 7A and B). Consistent with these results, the proportion of the root area colonized by the bacterium was significantly reduced in plants treated with the strong elicitor OG_DP10-15_ (9% of the total root system area) compared with OG_B_-treated or water-treated control plants (54% and 44%, respectively) (Fig. 7C). The colonization pattern of *B.v* along the root system was also impacted by OG_DP10-15_ treatment, resulting in a reduction of the area colonized on the primary root and a preferential establishment on lateral roots, in contrast to OG_B_ treatment and control conditions.(Fig. 7D and E and Supplementary Fig. S7). Additionally*,* the bacterium preferentially settled into thick localized biofilm-like structures on lateral roots in seedlings treated with OG_DP10-15,_ which contrasts with the prospective behavior observed through increased root system exploration by motile cells upon treatment with OG_B_ or control conditions (Fig. 7F and Supplementary Fig. S7). As a typical response of bacilli to stressful conditions [55], treatment with OG_DP10-15_ stimulated spore formation in the biofilm developing along the roots to a significantly greater extent than OG_B_ treatment, which induced only a moderate increase in spore population compared to control conditions (Fig. 7G).

## Discussion

Despite growing knowledge in the complex dialogue occurring between beneficial microorganisms and their host plants, we are only scratching the surface of the chemical diversity and molecular mechanisms employed by both partners to communicate. In this work, we show that the plant cell wall plays a pivotal role in this interkingdom crosstalk as the beneficial bacterium *B.v* actively interacts with pectin, a key structural component, to generate specific OGs with a low DP. These OGs exhibit a dual function in host immunity, by attenuating local immune responses to promote bacterial colonization of roots while simultaneously activating systemic resistance against foliar pathogens. Our results shed new light on the dynamic interplay between plant cell wall components and beneficial microbes, revealing a sophisticated strategy for immune modulation and microbial accommodation.

Deploying an arsenal of plant cell wall degrading enzymes, particularly those targeting pectin polymer, has long been considered as a feature of phytopathogenic micro-organisms [26]. These enzymes enable the release of carbohydrates from complex polymers such as pectin that can be used as nutrients and thereby, promote pathogen invasion and virulence [25, 26]. In contrast, *B.v* displays a very limited repertoire of pectin-degrading enzymes constituted of only two functional lyases PelA and PelB, which contribute to root colonization, suggesting a clear involvement in rhizosphere niche establishment [32]. Still, the role of these enzymes was unclear especially considering that, contrary to pathogens, *B.v* is unable to use pectin backbone as a carbon source due to an incomplete HG degradation pathway [32]. Pathogen infection is associated with the accumulation of high DP OGs *via* a complex mechanism involving plant proteins that bind fungal polygalacturonase (PGIPs), hindering further digestion into smaller fragments to activate the plant immune system and fight the infection [33]. Instead, our results reveal that *B.v* releases a specific low DP OGs profile *in planta* through the activity of its two lyases compared with OGs released upon pathogen ingress, suggesting that such defensive response of the plant is not triggered by the beneficial bacterium. This is further supported by our transcriptional analysis of the PGIP-encoding gene in the roots of OG_B_-treated seedlings, which showed no induction of the corresponding gene in contrast with OG_DP10-15_ treatment.

The DP of OGs is particularly relevant in the context of plant-microbe interactions, as it directly influences their potential to trigger immune responses. Most studies indicate that at least a DP_10-15_ is required for OGs to function as effective elicitors of plant defense responses [19, 21, 23], whereas shorter-chain OGs exhibit little to no eliciting effect [33, 46, 56, 57]. Our findings demonstrate that local immune responses in plant roots, including the expression of marker genes of early immune responses to OG _DP10-15,_ ROS_apo_ burst, and Ca^2+^ influx induced by OG_B_ are significantly weaker compared to those triggered by OG_DP10-15_. Ca^2+^ signatures in *Arabidopsis* roots were notably distinct depending on the DP profile of the OGs used as elicitor, where pathogen-associated OGs provoke rapid and transient increase in cytosolic Ca^2+^ whereas those from mutualistic *Bacillus* induce less intense responses. Ca^2+^ signatures were shown to vary according to the external stimuli perceived and should therefore encode specific information driving plants to respond adequately [58]. For instance, based on distinct Ca^2+^ signatures observed in plant roots in response to chitin-derived oligomers CO4, CO8, and mycLCO, plants can distinguish fungal molecules to initiate either symbiotic or immune responses [59]. It is therefore tempting to draw a parallel with our data and Ca^2+^ signaling upon pectin oligomers perception that may be used by the host plant to differentiate between undesired pathogenic organisms and mutualistic organisms such as *B.v*.

Plant immunity can be seen as a major obstacle that beneficial bacteria need to tackle during early colonization of the root surface. Our results unveil a distinctive behavior of *B.v* on the plant roots depending on the strength of the elicitor perceived by its host. Upon elicitation with OG_B_, the beneficial bacterium displays a prospective behavior, by exploring the entire root system under the form of motile cells during the initial attachment phase of biofilm-like structure formation. In contrast, treatment with OG_DP10-15_ results in a reduction of the exploration of the primary root by isolated cells and instead, induces the formation of disparate cell aggregates. Such early switch from motile cells to biofilm-like patches on the primary root upon OG_DP10-15_ elicitation might be seen as a strategy displayed by *B.v* to protect the bacterial community from toxic compounds released by the plant thanks to the shield created by exopolysaccharides and the hydrophobin layer of the biofilm structure [60–63]. Moreover, the mature biofilm that developed along the roots is significantly enriched in spores upon OG_DP10-15_ treatment in comparison with OG_B_, highlighting a clear survival mechanism deployed by *B.v* to face the adverse outcomes of triggering strong immune responses. Our data also indicate that elicitation of the primary root with OG_DP10-15_ triggers spatial evasion in *B.v.*, leading to a preferential colonization of lateral roots and a reduced total root area colonized whereas the bacterium efficiently colonizes the entire root system upon OG_B_ elicitation. This difference is particularly interesting given that robust root colonization capacity is closely associated with the potential of PGPRs to exert beneficial functions for their host plant [64].

The ability to subvert root immune responses is also a critical fitness determinant of all types of plant-associated bacteria being either pathogens, symbiotes, or non-symbiotic rhizobacteria. Flagellin of pathogens, especially the Flg22 epitope, is well known to induce strong PTI responses that hinder bacterial colonization and plant growth [65–67]. As other beneficials [16, 17, 67–69], bacilli have evolved strategies such as deployment of peptide variations in Flg22 epitope or secretion of immune-suppressive compounds to mitigate plant defense responses to their perception [54, 70]. Moreover, we previously showed that upon sensing the HG pectin backbone, a general repression of motility-related genes is induced in *B.v*, including those involved in flagellin synthesis, to initiate biofilm formation [32]. All these distinct mechanisms underscore the critical role of modulating plant immunity to facilitate the establishment of a mutualistic relationship with the host plant. Here, our data indicate that the interaction of pectin degradation products generated by *B.v* and the host plant roots results in a dampening of immune responses directed against the bacterium. This may contribute to create a favorable environment for the bacterium, ensuring the successful establishment of high *B.v* cell population harboring immunogenic MAMPs that can trigger strong immune reaction when perceived by the host. Consistent with this, we show here that the expression of *pel* genes in *B.v.* is stimulated by the perception of pectin backbone and is regulated by quorum sensing [32], which is activated at high cell density. We assume that such regulation, in turn, promotes the accumulation of OG_B_ by *B.v* in the vicinity of the plant root, further emphasizing the connection between pectin degradation products and niche establishment. It may explain the reduced colonization potential of the *ΔPelAΔPelB* mutant unable to generate such pectin fragments compared to the wild type strain. Thus, in contrast to pathogens, the beneficial bacterium might have co-evolved with its host plant to avoid triggering strong immune responses caused by partial degradation of pectin during root colonization, ensuring the preservation of a mutualistic relationship with its host. That being said, the potential to generate OGs *via* Pel enzymes might not be restricted to the species *B. velezensis* because the corresponding genes are conserved in other plant-associated bacilli [32]. Furthermore, given the conserved nature of pectin across plant species, this OGs-mediated interaction may extend beyond the system tomato/*Arabidopsis*-*B.v* studied here. However, whether other taxa of plant-associated beneficial rhizobacteria express functional pectinases encoded by *pel* genes remains to be determined.

The advantages of this interaction mediated through OG_B_ are not limited to bacterium, as it also confers higher systemic resistance to the plant towards infection by pathogens with different lifestyles (i.e. necrotrophic and hemibiotrophic) and therefore that trigger plant immunity through distinct pathways [71]. So far, ISR induced by beneficial bacilli was primarily attributed to secondary metabolites including cyclic lipopeptides (surfactin and to a lesser extent iturin and fengycin) [11–13] and volatile compounds (acetoin and 2,3-butanediol) [72–74]. Here, we unveil short HG fragments as a novel class of infochemicals mobilized by *B.v* to enhance the host defense potential. Plant leaf spraying with OG_DP10-15_ was commonly reported to enhance local resistance across various pathosystems [75–80]. However, protection was often accompanied by a growth cost [53, 75, 81, 82]. Our data illustrate that OG_B_ perception at the root level results in a limited PTI-like response without causing significant growth cost and efficiently primes the plant for robust systemic resistance against pathogen ingress. Therefore, as hallmark of a beneficial interaction, OG_B_ potentiate resistance through a process that does not impair the fitness of its host. Still, the molecular mechanisms underlying the perception of OGs with varying DP by plant cells remain unclear. It was proposed that high DP OGs need to assemble in egg-box like structures through Ca^2+^-mediated intermolecular cross-links, to be recognized by the WAK1 receptor and initiate immune signaling [83]. However, short OGs do not form such supra molecular structures but are still immunogenic albeit to a lesser extent [33, 46, 56, 57]. Moreover, a recent study showed that none of the five WAK-type receptors potentially involved in OGs perception (WAK1-5) was required for immune activation triggered either by long or short non-methylesterified OGs [75]. Thus, the precise mechanism by which OGs are perceived, allowing the plant to distinguish friends from foes through the detection of different type of pectin fragments remains to be fully elucidated.

In conclusion, by generating short-chain OG_B_ that both dampen localized immune reactions and induce systemic resistance, *B.v* exemplifies a strategy by which non-pathogenic rhizobacteria can circumvent robust plant immune defenses to establish and maintain a reciprocal interaction with its host. This interaction promotes the fitness of both partners by ensuring a safe root environment for bacterial colonization and by strengthening host resilience against pathogens without growth cost. These findings provide new insights into how specific bacterial enzymatic activities can modulate plant-derived signals to promote mutualistic associations. It should be emphasized that multiple other signal molecules displayed or secreted by the bacterium such as MAMPs and secondary metabolites respectively have been identified as involved in the molecular cross talk and so their combined integration could be crucial for plants to recognize *B.v* as a beneficial partner rather than a threat. Ultimately, exploring the molecular perception and downstream signaling pathways involved in plant-rhizobacteria interactions *via* cell wall-derived signals could also provide promising strategies for developing sustainable microbe-based approaches to improve crop health and protection. This includes the combination of beneficial bacteria and crude cell wall polymers generated as by-products of industries such as textile, paper, and food processing or the direct application of low-PTI-inducing cell wall fragments generated by beneficials, such as OG_B_.

## Supplementary Material

Supplementary_data_Boubsi_et_al_revised_wraf232

Video_S1_wraf232

Video_S2_wraf232

Video_S3_wraf232

## Data Availability

All data generated or analyzed during this study are included in the paper and/or in the Supplementary files.
