# Supplementary material for: Pectin-derived oligogalacturonides shape mutualistic interactions between Bacillus and its host plant
Source: ISME J. 2025 Oct 18;19(1):wraf232. doi: 10.1093/ismejo/wraf232 (PMC12598631; doi:10.1093/ismejo/wraf232)
Supplement: Supplementary_data_Boubsi_et_al_revised_wraf232 [file supplementary_data_boubsi_et_al_revised_wraf232.docx]

**Pectin-derived oligogalacturonides shape mutualistic interactions between *Bacillus* and its host plant**

Farah Boubsi^1*^, Adrien Anckaert^1^, Anthony Argüelles-Arias^1^ and Marc Ongena^1,2*^

*^1^ Microbial Processes and Interactions, TERRA Teaching and Research Center, University of Liège ‐ Gembloux Agro‐Bio Tech, Gembloux, 5030, Belgium*

^2^ Lead contact

^*^Correspondence : [farah.boubsi@uliege.be](mailto:farah.boubsi@uliege.be), [marc.ongena@uliege.be](mailto:marc.ongena@uliege.be)

Key words: *Bacillus velezensis*, plant cell wall, oligogalacturonides, pattern-triggered immunity, induced systemic resistance, plant protection, colonization, mutualism

**Supplementary data**

**
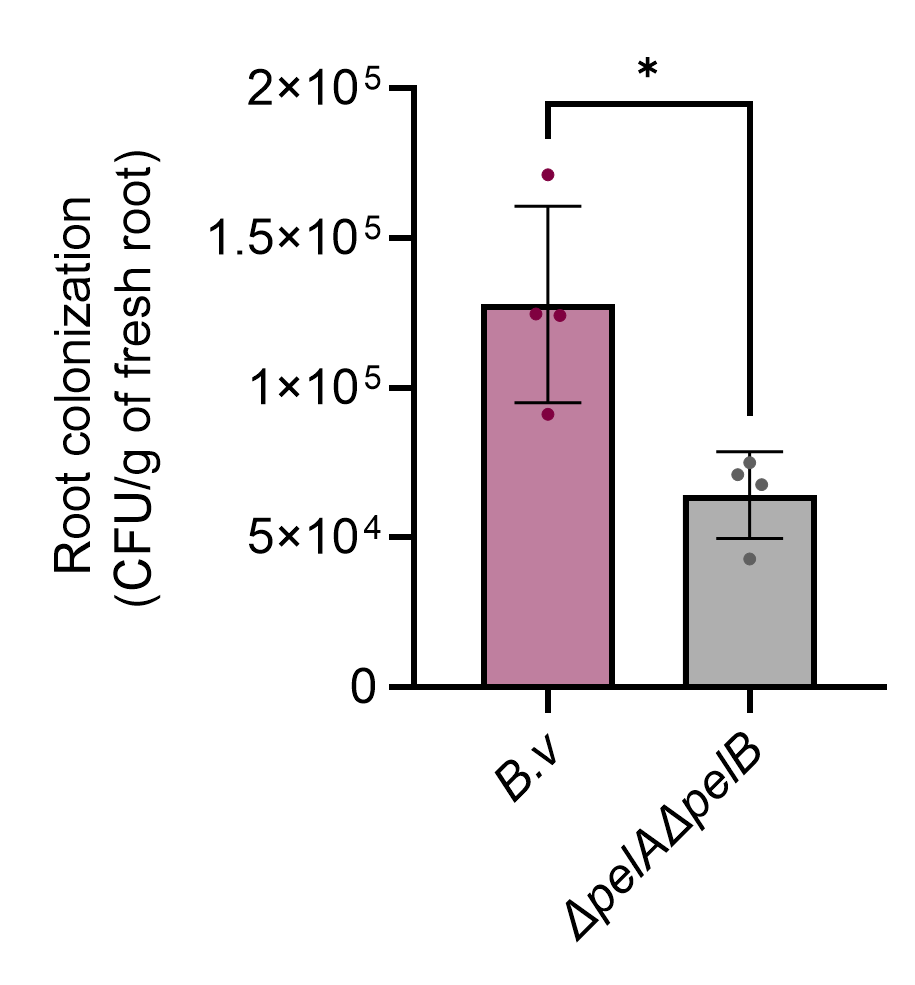
**

**Figure S1. Quantification of the total population of mCherry-tagged *B.v* and the mutant strain *ΔpelAΔpelB* on tomato roots 7 days post-inoculation by plate counting**. Results are expressed in terms of colony forming units (CFU) per gram of root fresh weight (Mean ± SD ; n=4 biological replicates; t-test; *, P<0.05).

**
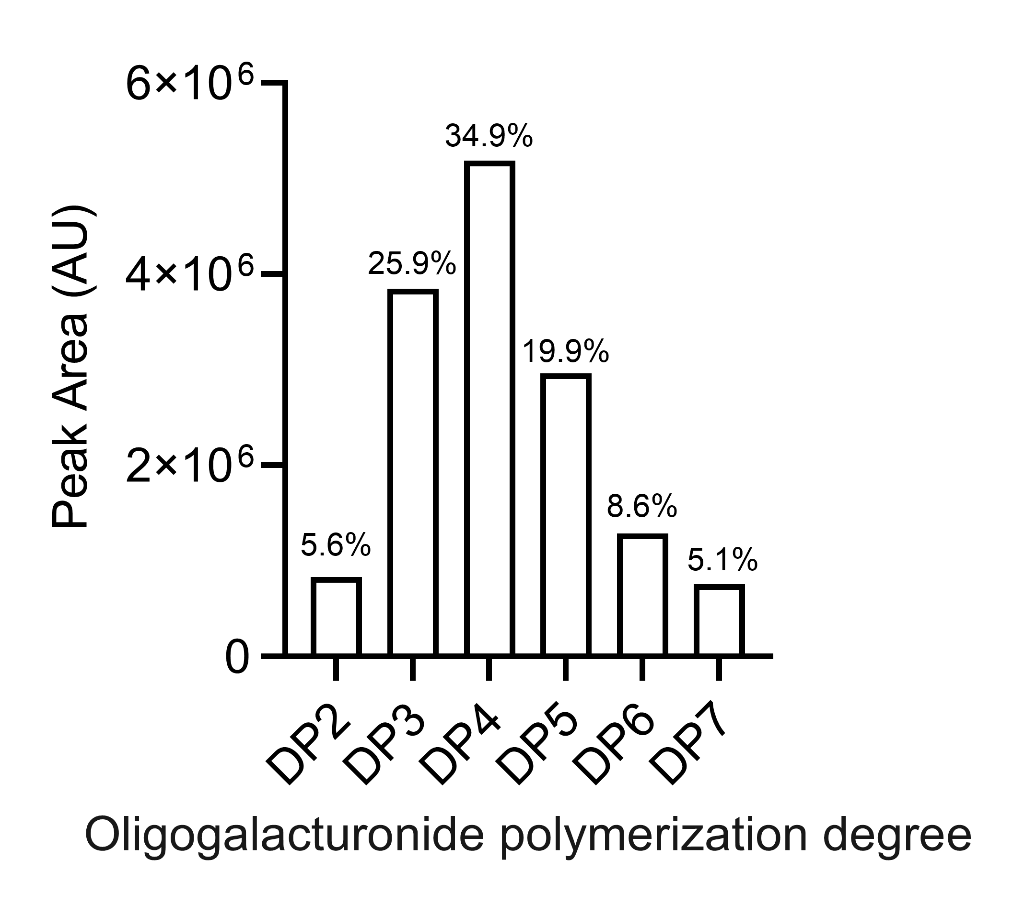
**

**Figure S2. UPLC-qTOF-MS characterization of oligogalacturonides produced by the activity of PelA-PelB enriched extract originating from a cell-free culture supernatant of *B.v* on commercial homogalacturonan (HG).** Relative amount of each DP detected is indicated above its corresponding bar.


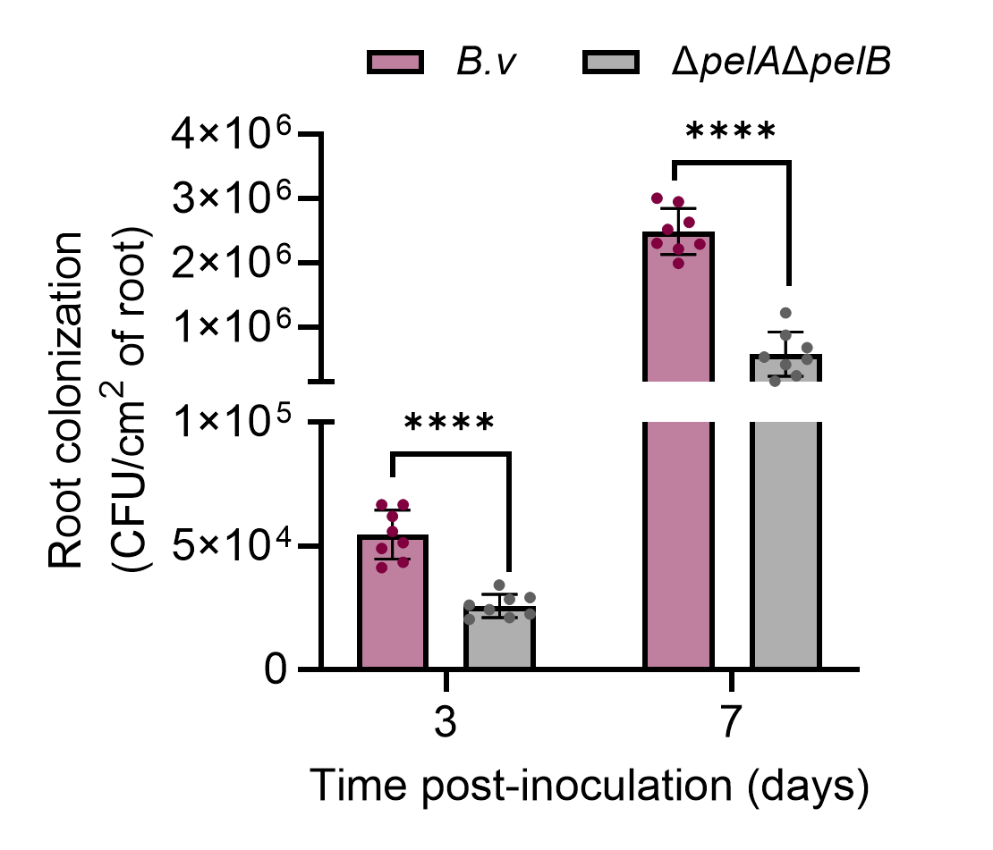


**Figure S3. Quantification of *Arabidopsis* root colonization over time (3 and 7 days post-inoculation) by *B.v* and the mutant strain *ΔpelAΔpelB*.** Quantification was performed by plate counting and results are expressed in terms of colony forming units (CFU) per cm^2^ of root (Mean ± SD; n=8 biological replicates, 2 independent experiments; t-test; ****, *P*<0.0001).

**
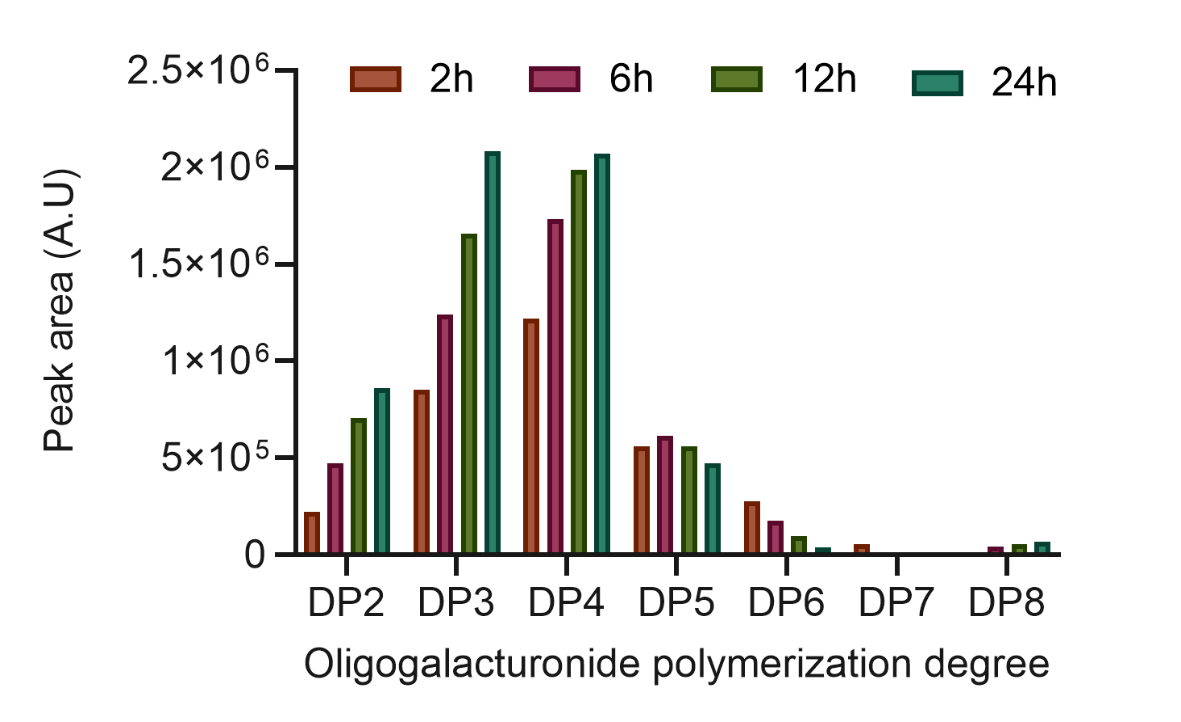
**

**Figure S4. UPLC-qTOF-MS profiling of oligogalacturonides produced over time from *Arabidopsis* roots by the activity of cell free PelA-PelB enriched extract from a culture supernatant of *B.v*.**

**
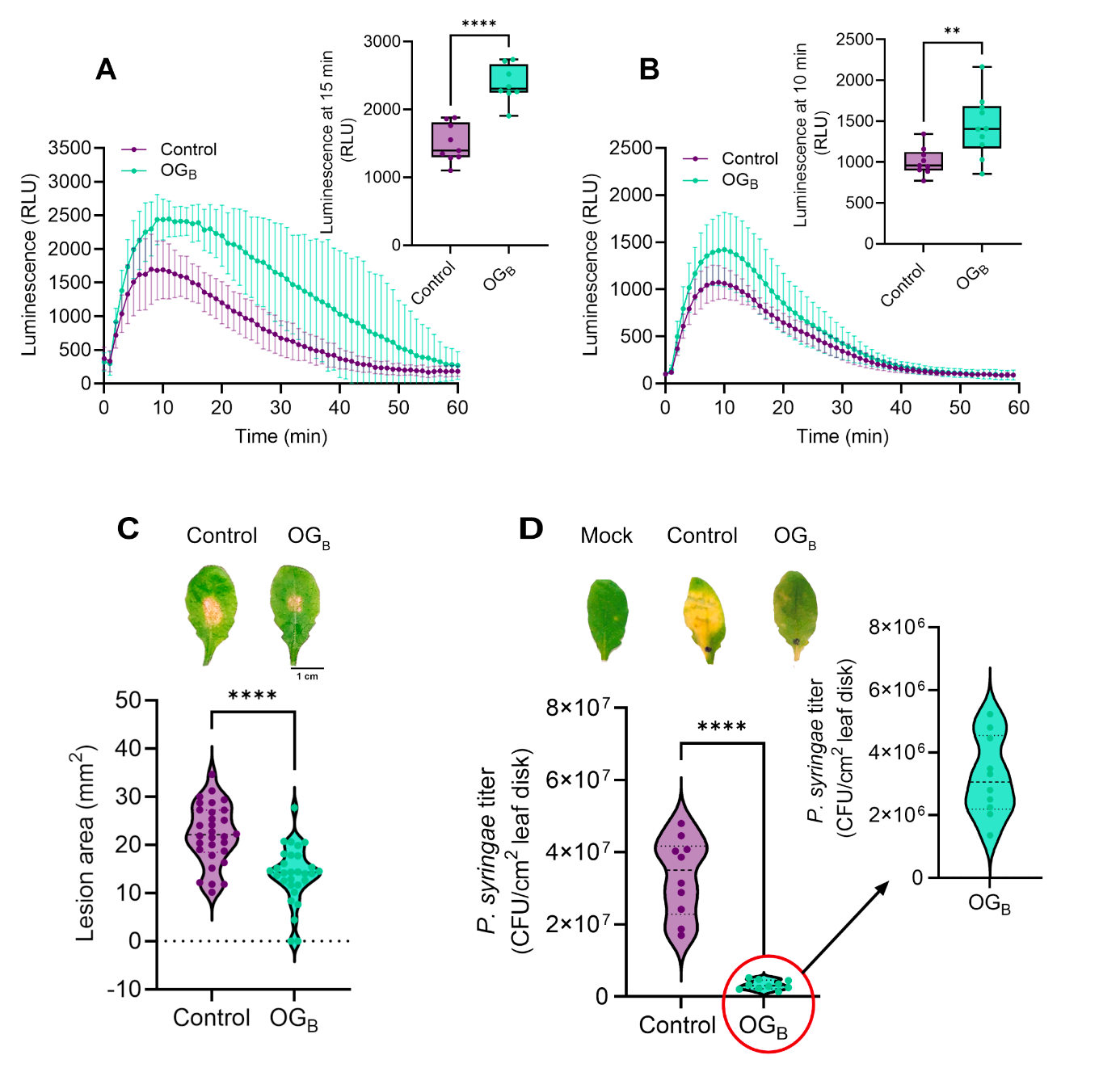
**

**Figure S5. OG_B_ activate systemic immune response and resistance in *Arabidopsis*.** (A and B) SIA assay on leaves of 4-week-old *Arabidopsis* plants whose roots were pretreated or not with 50 µg/ml OG_B_. Leaf disks were either elicited with 100 µg/ml chitin (A) or 1 µM Flg22 (B) (Mean ± SD; 8≤n≤10 biological replicates with one disk per individual plant, 2 independent experiments). Boxplots show the quantification of the maximal ROS_apo_ production measured by chemiluminescence in each condition (t-test; **, P<0.01; ****, P<0.0001) and encompass the 1^st^ and 3^rd^ quartiles, the whiskers extend to the minimum and maximum points, and the midline indicates the median. (C) Quantification of the lesion area observed on leaves of *Arabidopsis* plants whose roots were pretreated or not with 50 µg/ml OG_B_ 5 days post-infection with a spore suspension of *B. cinerea* (5x10^5^ spores/ml) (n=30, 3 leaves infected per plant from 10 individual plants; t-test; ****, *P*<0.0001). Images are representative pictures of spreading lesions observed on *Arabidopsis* leaves 5 days post-infection with *B. cinerea*. (D) Bacterial titer of *P. syringae* pv. tomato DC3000 on leaves of *Arabidopsis* plants whose roots were pretreated or not with 50 µg/ml OG_B_ 3 days post-infiltration (n=10, 3 leaves infected per plant from 10 individual plants; t-test; ****, *P*<0.0001). Images are representative pictures of OG_B_-induced resistance of *Arabidopsis* plants against *P. syringae* pv. tomato DC3000 3 days post-infiltration. All violin plots of the panel show the distribution and density of the data. The width of the violin indicates the density at different values, with thicker sections representing higher data density. The central bold dashed line represents the median and the thin dashed lines indicate the interquartile range.


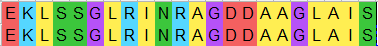


*Bacillus subtilis* 168

*Bacillus velezensis* GA1

**Figure S6. Amino acid sequence alignment of the Flg22 epitope of *B. subtilis* 168 and *B. velezensis* GA1** **using Mega11 software [1].**

**
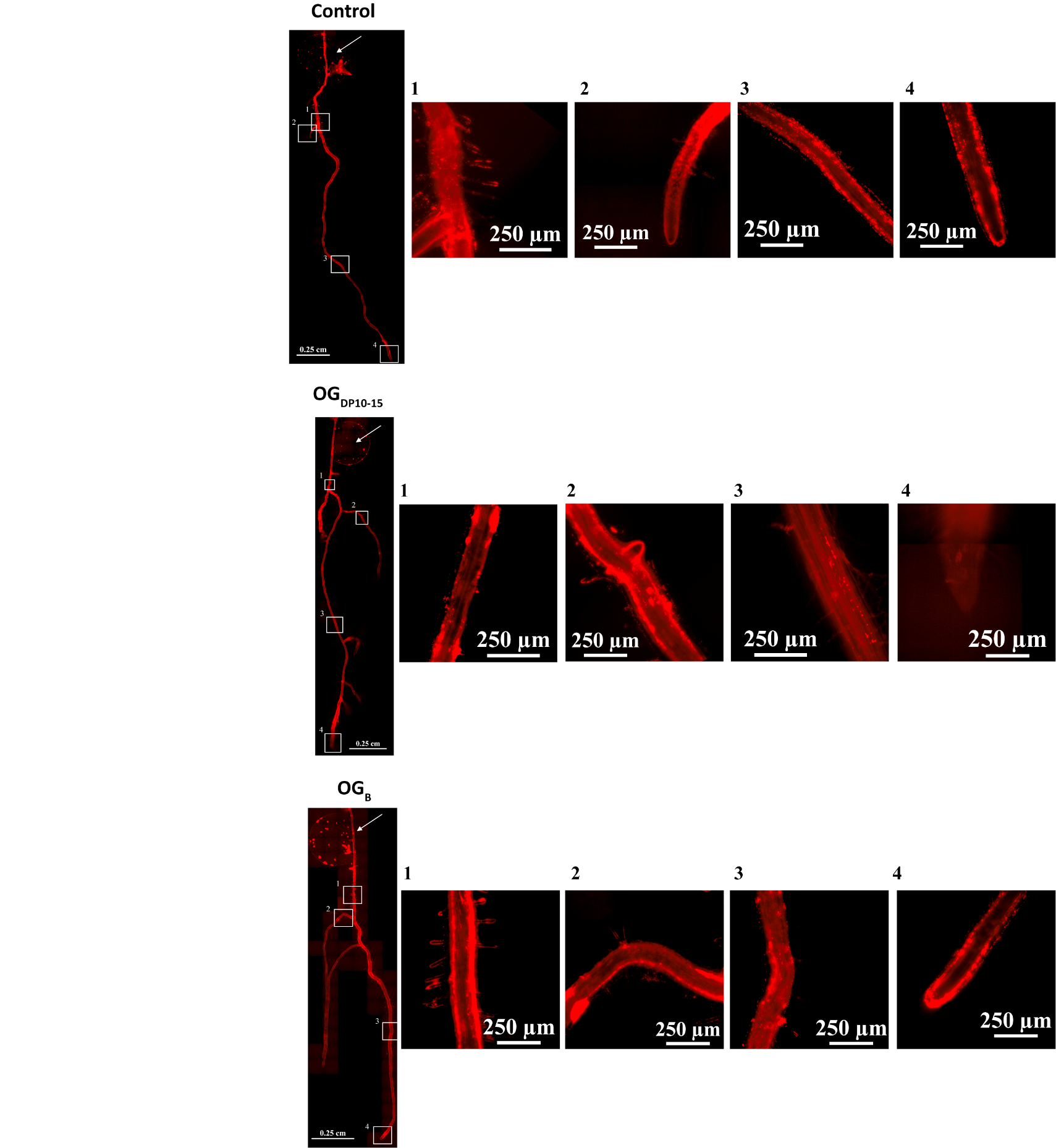
Figure S7.** **Microscopic composite pictures of mCherry-tagged *B.v* colonization along roots of *Arabidopsis* plantlets elicited at the root tip with water as control, 50 µg/ml OG_DP10-15_, or 50 µg/ml OG_B_**. Images were taken 2 days post-inoculation. White arrow indicate the inoculation drop of the bacterial suspension and numbered white squares delimit the different zones of the root from which detailed microscopic pictures were captured.


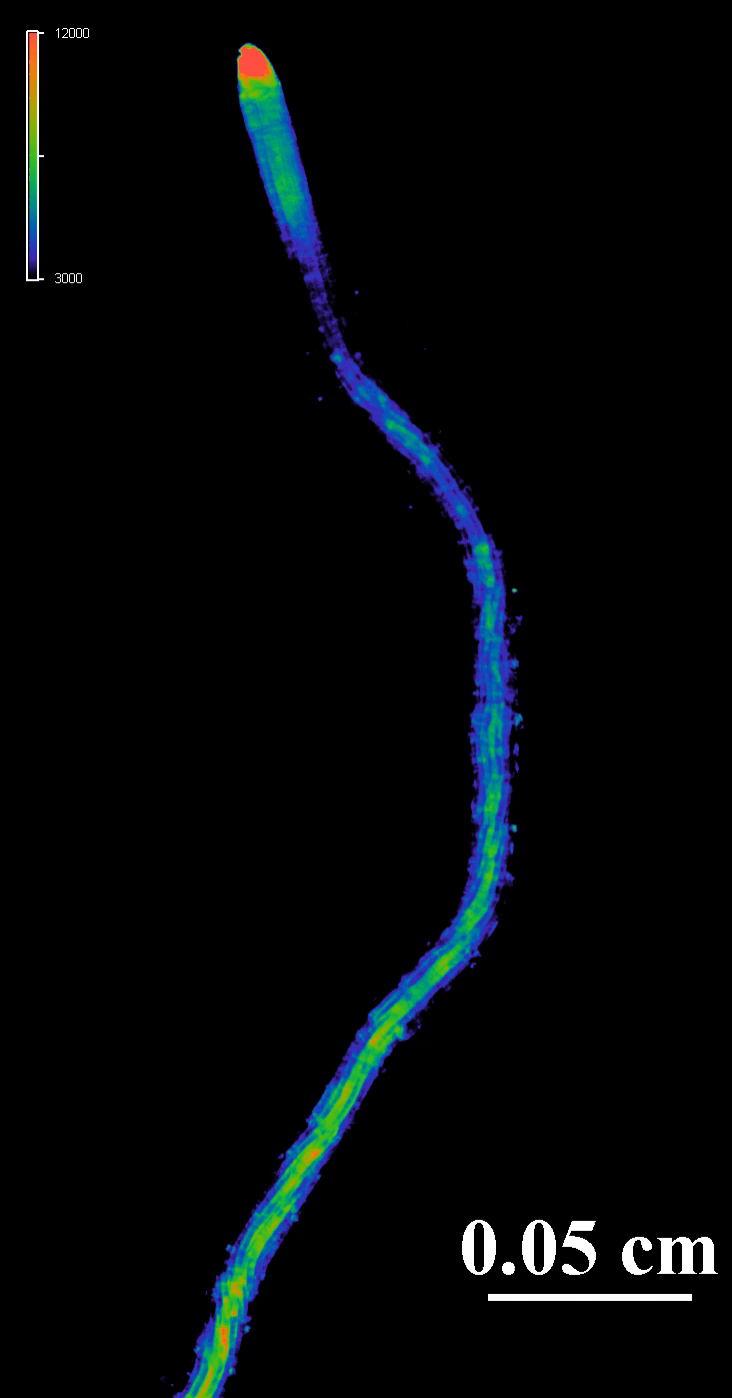
 Control OG_DP10-15_ OG_B_


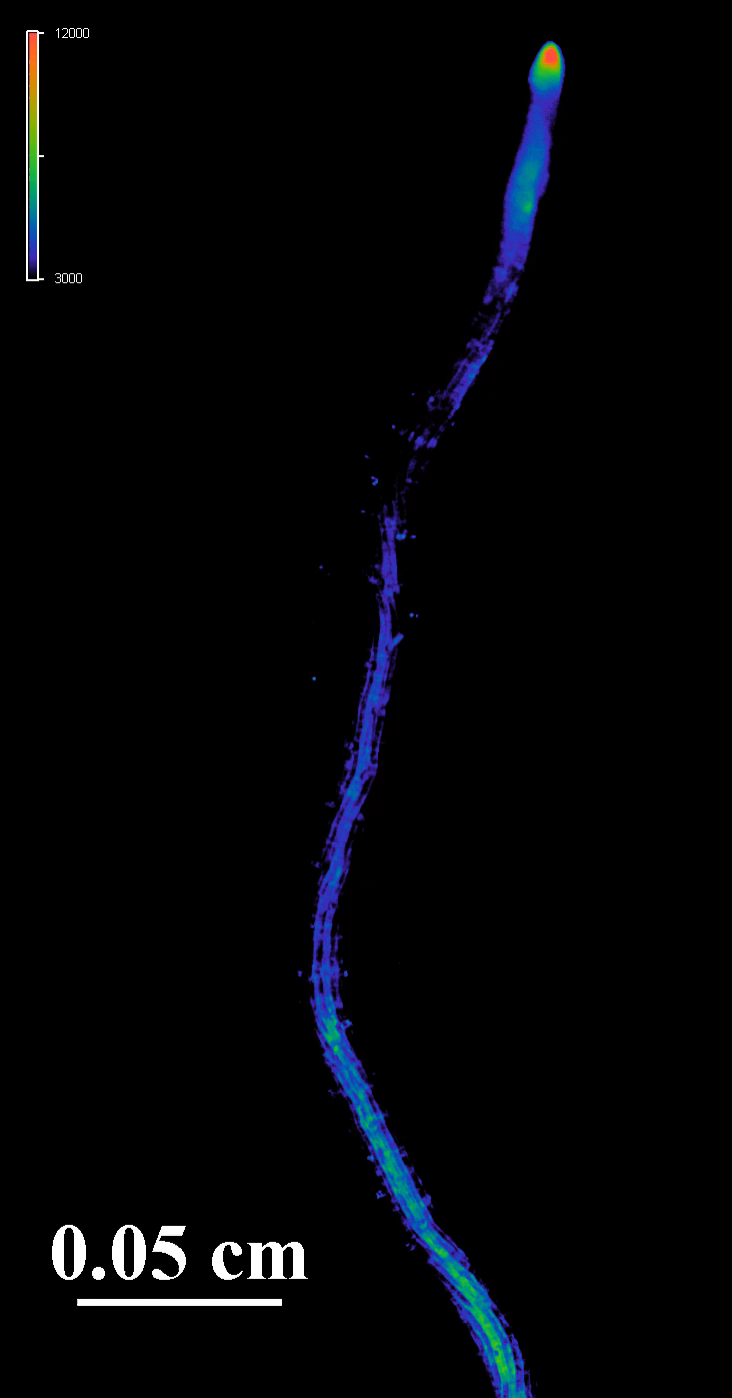

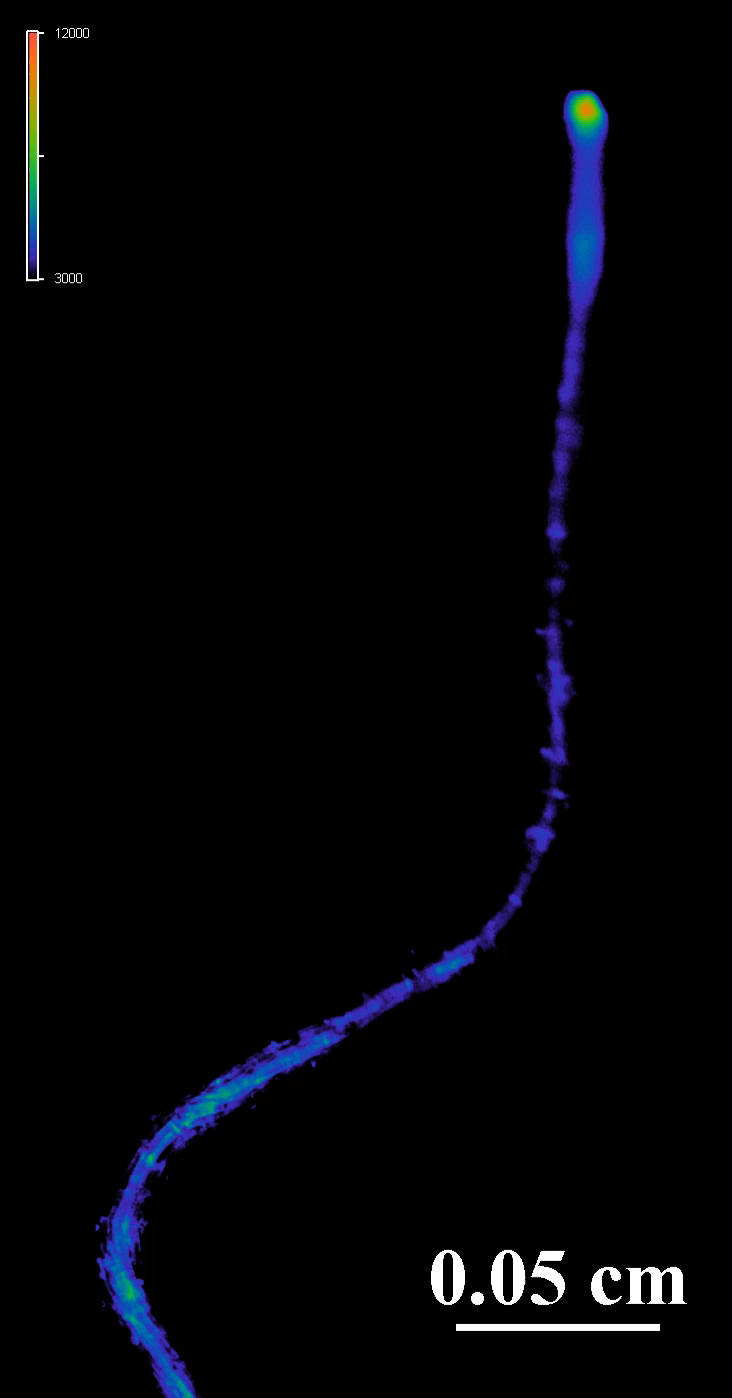


**Videos S1, S2 and S3. Pseudo-coloured live imaging of changes in [Ca^2+^]_cyt_ in the roots of *Arabidopsis* UBQ10::GCaMP3 reporter line upon elicitation at the root tip with water as control (S1), 50 µg/ml OG_DP10-15_ (S2) or 50 µg/ml OG_B_ (S3).**

**Supplementary methods**

**Quantification of *B.v* colonization on tomato roots**

To quantify the root colonization of tomato plants by *B.v* mCherry-tagged from SIA assay, 1 g of roots were cut into small pieces and placed in tubes containing glass beads and 5 ml of a solution of PBS+0.2% (w/v) Tween20. Tubes were vortexed vigorously for 5 min to tear off the bacterial cells from the roots. The total colony-forming units (CFUs) were determined by plating serial dilutions of the bacterial solutions on solid LB medium supplemented with 5 µg/ml chloramphenicol. Plates were incubated overnight at 30°C before proceeding to CFUs counting. Results were expressed as CFU/g of fresh roots.

**Quantification of *B.v* and *ΔpelAΔpelB* mutant colonization on *Arabidopsis* roots**

Two-week-old *Arabidopsis* seedlings were grown in square Petri dishes filled with sterile MS_1/2_ supplemented with 1% (w/v) Bacto agar (Sigma, United States) and 0.01% (w/v) MES (Sigma, United States). Seedlings were inoculated with 3 µl (OD_600_ 0.1) of washed cells of *B.v* or its mutant *ΔpelAΔpelB* originating from an overnight preculture in RE_1/2_ medium. Three and four days post-inoculation, roots were separated from the aerial part and placed in Eppendorf tubes containing 1 ml PBS supplemented with 0.1% (w/v) Tween20. Tubes were vortexed for 5 min and bacterial suspensions were diluted in 1:10 series. Dilutions were then plated on solid LB (for *B.v*) or solid LB supplemented with 5 µg/ml chloramphenicol (for *ΔpelAΔpelB* mutant) and incubated at 30°C overnight for colony counting. Bacterial population was normalized against the corresponding seedling total root area and results were expressed as CFU/cm^2^ of root.

**ISR against *Botrytis cinerea* in *Arabidopsis***

One-week-old seedlings were transferred to seed holders of Araponics systems (Araponics, Belgium) filled with 0.7% agar (w/v) and were grown hydroponically for 3 weeks in nutrient solution (0.25% (v/v) FLORAMICRO, 0.25% (v/v) FLORABLOOM, 0.25% (v/v) FLORAGRO; General Hydroponics). Plants were then placed individually for one additional week into 50 ml falcons covered with aluminum foil containing the same solution. Roots were then pretreated overnight with OG_B_ (50 µg/ml) or nutrient solution as control. *Botrytis cinerea* strain MUCL 43839 was cultured on solid PDA for 15 days in the dark at room temperature. Spores were then collected in a germination solution (1.75 g/l KH_2_PO_4_, 0.74 g/l MgSO_4_, 4g/l glucose, 0.02% (v/v) Tween20), filtered through a sterile gauze, quantified, and adjusted to a concentration of 5x10^5^ spores/ml. The spore suspension was then incubated overnight under shaking at 26°C to allow the pre-germination of the spores. Infection was performed on detached leaves as previously described [2] by applying a 5 µl drop of *B. cinerea* spore suspension onto the fourth to the sixth leaf of each individual plant. Infected leaves were incubated in Petri dishes filled with agar 1% (w/v) at 19°C with a HR of 65% under a 16 h/8 h day/night cycle (100 µmol s^-1^ m^-2^). The area of spreading lesions was measured by color thresholding using ImageJ Fiji software [3] 5 days post-infection.

**TableS1. List of RT-qPCR primers used in this study.**

| **Targeted gene** | **Primer name** | **Primer Sequence (5’→3’)** | **Reference** |
| --- | --- | --- | --- |
| ***Bacillus velezensis* GA1** | | | |
| *pelA* | pelAFw | GACGGCAGACGTTAGGAACA | This study |
|  | pelARv | ACGAGCTGCTGTCTGTTTGA |  |
| *pelB* | pelBFw | ACCGGGCCTGATGAGATACG | This study |
|  | pelBRv | GGCTTTGAGCGACGGGAATG |  |
| *gyrA* | gyrAFw | GAGACGCACTGAAATCGTGA | This study |
|  | gyrARv | GCCGGGAGACGTTTAACATA |  |
| ***Arabidopsis thaliana* Col-0** | | | |
| *RBOHD* | rbohDFw | CGAATGGCATCCTTTCTCAATC | [4] |
|  | rbohDRv | GTCACCGAGAGTGCGGATATG |  |
| *CML41* | cml41Fw | CCGACGAAGATCACCAAAAT | [5] |
|  | cml41Rv | TGTCTGAGCTCAAAGGCTGA |  |
| *WRKY40* | wrky40Fw | GATCCACCGACAAGTGCTTT | [5] |
|  | wrky40Rv | AGGGCTGATTTGATCCCTCT |  |
| *PER4* | per4Fw | GGAACCGGAGCTTCTACAGATAG | [5] |
|  | per4Rv | TTAATTAACGGCACTGCAGATTC |  |
| *CYP81F2* | cyp81F2Fw | GTGAAAGCACTAGGCGAAGC | [5] |
|  | cyp81F2Rv | ATCCGTTCCAGCTAGCATCA |  |
| *PGIP1* | pgip1Fw | GACGAATCTGACAGGTCCAA | [5] |
|  | pgip1Rv | ATAGGCGAAGGTCAGGGACT |  |
| *UBQ10* | ubq10Fw | GGCCTTGTATAATCCCTGATGAATAAG | [5] |
|  | ubq10Rv | AAAGAGATAACAGGAACGGAAACATAGT |  |
| *ACT7* | act7Fw | GGTGTCATGGTTGGTATGGGTC | [5] |
|  | act7Rv | CCTCTGTGAGTAGAACTGGGTGC |  |
| *TUB6* | tub6Fw | GAAGTCMGCGTCTGTG | [5] |
|  | tub6Rv | ACTCGCCTTCGTCATC |  |
| *EF1Α* | ef1αFw | TCCAGCTAAGGGTGCC | [5] |
|  | ef1αRv | GGTGGGTACTCGGAGA |  |
| *EIF4A* | eif4AFw | ACTGACCTCTTAGCTCG | [5] |
|  | eif4ARv | CAGATCGGCCACGTTC |  |
| *UBQ5* | ubq5Fw | GGAAGAAGAAGACTTACACC | [6] |
|  | ubq5Rv | AGTCCACACTTACCACAGTA |  |

**Supplementary references**

1. Tamura K, Stecher G, Kumar S. MEGA11: Molecular Evolutionary Genetics Analysis Version 11. *Mol Biol Evol* 2021;**38**:3022–7, DOI: 10.1093/molbev/msab120.

2. Ingle RA, Roden LC. Circadian Regulation of Plant Immunity to Pathogens. In: Staiger, D. (eds.) *Plant Circadian Networks*. *Methods in Molecular Biology*. New York, NY: Humana Press, 2014, 273–83, DOI: 10.1007/978-1-4939-0700-7_18

3. Schindelin J, Arganda-Carreras I, Frise E *et al.* Fiji: an open-source platform for biological-image analysis. *Nat Methods* 2012;**9**:676–82, DOI: 10.1038/nmeth.2019.

4. Morales J, Kadota Y, Zipfel C *et al.* The Arabidopsis NADPH oxidases RbohD and RbohF display differential expression patterns and contributions during plant immunity. *J Exp Bot* 2016;**67**:1663–76, DOI: 10.1093/jxb/erv558.

5. Davidsson P, Broberg M, Kariola T *et al.* Short oligogalacturonides induce pathogen resistance-associated gene expression in Arabidopsis thaliana. *BMC Plant Biol* 2017;**17**:19, DOI: 10.1186/s12870-016-0959-1.

6. Lorrai R, Francocci F, Gully K *et al.* Impaired Cuticle Functionality and Robust Resistance to Botrytis cinerea in Arabidopsis thaliana Plants With Altered Homogalacturonan Integrity Are Dependent on the Class III Peroxidase AtPRX71. *Front Plant Sci* 2021;**12**:696955, DOI: 10.3389/fpls.2021.696955.
